# Comprehensive Urinary Bile Acid Profiling in Patients Investigated for Inborn Errors of Bile Acid Synthesis Using LC–MS/MS

**DOI:** 10.3390/metabo16100753

**Published:** 2026-10-08

**Authors:** Ufuk Bozkurt Obuz, Duygu Demirtaş Güner, Ersin Gümüş, Basri Gülbakan, Hülya Demir, Hasan Özen, Turgay Coşkun, İncilay Lay

**Affiliations:** 1 Department of Medical Biochemistry, Faculty of Medicine, Hacettepe University, 06100 Ankara, Türkiye; lincilay@gmail.com; 2 Scientific and Technological Research Center, Sinop University, 57000 Sinop, Türkiye; 3 Department of Pediatrics, Division of Gastroenterology, Hepatology and Nutrition, Faculty of Medicine, Hacettepe University, 06100 Ankara, Türkiye; duygudemirtas@gmail.com (D.D.G.);; 4 Department of Pediatric Basic Sciences, Institute of Child Health, Hacettepe University, 06100 Ankara, Türkiye; 5 Department of Pediatrics, Division of Pediatric Metabolism and Nutrition, Faculty of Medicine, Hacettepe University, 06100 Ankara, Türkiye; 6 Clinical Chemistry Laboratory, Hacettepe University Hospitals, 06100 Ankara, Türkiye

**Keywords:** bile acids, inborn errors of bile acid synthesis, targeted bile acid profiling, tandem mass spectrometry, cholestatic liver disease

## Abstract

Background/Objectives: This study examined urinary cholanoid profiles in a descriptive case series evaluated for suspected inborn errors of bile acid synthesis (IEBAS) or unexplained cholestasis. Methods: Urinary excretion of 66 bile acid metabolites was measured using liquid chromatography–tandem mass spectrometry (LC-MS/MS), and concentrations were interpreted as semi-quantitative estimates. In addition, cholesterol, cholestanol, and 7-dehydrocholesterol were analyzed by gas chromatography–mass spectrometry (GC-MS). Results: Five patients had genetically confirmed IEBAS: 3β-hydroxy-Δ5-C27-steroid dehydrogenase (HSD3B7) deficiency in 2 patients and cerebrotendinous xanthomatosis (CTX) in 3 patients. Two patients were subsequently diagnosed with biliary atresia and progressive familial intrahepatic cholestasis type 1. Other patients remained without a definitive etiological diagnosis. Of the two patients with genetically confirmed HSD3B7 deficiency, patient 1 showed a predominance of 3β-hydroxy-Δ^5^ bile acids (63.9% of total urinary bile acids), including sulfated dihydroxy-, glycine-conjugated dihydroxy-, and trihydroxy-Δ^5^ species. Patient 2 had an atypical urinary bile acid profile, with the 3β-hydroxy-Δ^5^ fraction dominated by a monohydroxy species. CTX patients had profiles with a different bile acid group composition; hydroxylated bile acids were reported in 2 patients, whereas no numerical result was reported for 1 patient. The 3β-hydroxy-Δ^5^ bile acid levels in 1 CTX patient were within the observed control range. Two patients also had differential profiles for hydroxylated and 3β-hydroxy-Δ^5^ bile acids, which provided biochemical findings for further etiological evaluation. The species assigned to glycoursodeoxycholic acid predominated in the urinary profiles of the five patients receiving ursodeoxycholic acid. Conclusions: Urinary cholanoid profiling may provide complementary biochemical information alongside the available clinical and genetic findings in the evaluation of suspected IEBAS.

## 1. Introduction

Patients with progressive cholestatic liver disease of unknown etiology should be evaluated for inborn errors of bile acid synthesis (IEBAS), as these rare metabolic disorders represent potentially treatable causes of otherwise unexplained cholestasis. These disorders result from pathogenic genetic variants that affect enzymes responsible for primary bile acid synthesis. Bile acid biosynthesis is a complex process involving at least 17 hepatic enzymes located in the endoplasmic reticulum, mitochondria, cytoplasm, and peroxisomes, with extensive trafficking of intermediates between these subcellular compartments [1]. Four major pathways of bile acid biosynthesis have been identified, namely the classical or neutral pathway, the alternative or acidic pathway, the Yamasaki pathway, and the 25-hydroxylation pathway. However, the precise order of reactions within these biosynthetic pathways remains unclear, as many intermediates can serve as substrates for more than one enzyme [2,3]. To date, 12 inherited metabolic disorders have been recognized as inborn errors of bile acid synthesis (IEBAS). These include Cerebrotendinous Xanthomatosis (CTX), 3β-hydroxy-Δ^5^-C27-steroid Dehydrogenase/Isomerase Deficiency (HSD3B7), Δ^4^-3-oxosteroid-5β-reductase Deficiency, Oxysterol 7α-hydroxylase Deficiency, Cholesterol 7α-hydroxylase Deficiency, and several defects localized in peroxisomes, namely α-methylacyl-CoA Racemase (AMACR) Deficiency, Acyl-CoA Oxidase 2 Deficiency, D-bifunctional Protein Deficiency, ATP-binding Cassette Subfamily D Member 3 Deficiency, Sterol Carrier Protein X (SCPx) Deficiency, Bile acid-CoA:amino acid N-acyltransferase deficiency, and Bile acid-CoA ligase deficiency [4].

Early diagnosis and appropriate treatment are crucial to prevent liver failure and associated neurological symptoms [5,6,7]. The characteristic clinical presentation of patients with IEBAS includes cholestasis, hepatomegaly, pale stools, elevated serum direct bilirubin levels, and normal gamma-glutamyltransferase and total bile acid levels. Defective enzyme function leads to overproduction of hepatotoxic atypical bile acids derived from accumulating metabolic intermediates, along with a deficiency of primary bile acids, resulting in impaired bile flow [8]. When IEBAS is suspected, mass spectrometry is considered the most appropriate screening method for detecting bile acid intermediates in plasma and urine [9,10,11,12].

In this study, urinary excretion profiles of 66 different bile acid species were investigated by LC–MS/MS in 12 patients evaluated for suspected IEBAS or unexplained cholestasis and in 50 healthy controls. Classical, hydroxylated, 3β-hydroxy-Δ^5^, 3-oxo-Δ^4^ and short- and long-chain bile acids were analyzed in dried urine spots. In addition, plasma cholesterol, 7-dehydrocholesterol and 5α-cholestanol were analyzed by GC–MS. The aim was to characterize urinary cholanoid profiles and assess their relationship with the available clinical and genetic findings.

## 2. Materials and Methods

### 2.1. Patient Characteristics and Sample Collection

This descriptive case series included 12 patients who met at least one of the following inclusion criteria: clinical suspicion of an IEBAS or unexplained cholestasis. Eligible patients were enrolled consecutively between September 2018 and September 2019. Patients 3–5 were investigated for clinically suspected cerebrotendinous xanthomatosis (CTX) despite routine liver biochemical parameters being within reference ranges. Definitive etiological diagnoses had not been established at enrollment or at the time of urinary bile acid profiling. The diagnoses reported for patients 1–5, 8, and 9 were established during subsequent clinical and/or genetic investigations.

The control group comprised 50 healthy volunteers without evidence of chronic liver disease. Controls were not age-matched and served as a descriptive comparison group; age-specific reference intervals for urinary bile acids were not established. Urine samples from patients and controls were collected and stored at −80 °C until analysis. Plasma samples were used for measurement of cholesterol, 7-dehydrocholesterol, and 5α-cholestanol. Urinary bile acid profiles were assessed cross-sectionally. Reference intervals for ALT, AST, GGT, direct bilirubin, and total bilirubin were obtained from the respective clinical laboratory reports and applied individually, as presented in Table 1.

The study was conducted in accordance with the Declaration of Helsinki (as revised in 2013) and was approved by the Ethics Committee of Hacettepe University (approval no. GO-18/255-04; 1 September 2018). Written informed consent was obtained from all study participants or their parents/legal guardians, as applicable.

Patient 1: The 8-year-old male patient was born to consanguineous parents. He presented at 40 days of age with jaundice, intramuscular bleeding following an antipyretic injection, and hepatomegaly. At 7 months of age, laboratory evaluation revealed elevated aspartate aminotransferase (AST), alanine aminotransferase (ALT), total and direct bilirubin, activated partial thromboplastin time (aPTT), and international normalized ratio (INR), with normal gamma-glutamyltransferase (GGT) levels. A liver biopsy showed giant cell transformation of hepatocytes, marked cholestasis, and pericellular fibrosis. Histopathological findings were reported as consistent with neonatal hepatitis or bile duct paucity, provided that extrahepatic biliary atresia was excluded. The distribution of cholestasis was not described, and the fibrosis stage was not specified in the biopsy report. At 4 years of age, the patient was admitted to our hospital with cirrhosis and portal hypertension, and jaundice improved following ursodeoxycholic acid (UDCA) treatment. Genetic analysis revealed no pathogenic variants in ATP8B1, ABCB11, or ABCB4 genes, associated with progressive familial intrahepatic cholestasis types 1–3 (PFIC1–3), respectively. UDCA treatment was discontinued 2 weeks prior to sample collection. The recorded UDCA regimen was 15 mg/kg/day (Table 2).

Patient 2: The 15-year-old male patient was born to consanguineous parents. He was admitted to the hospital at 2 months of age with jaundice, dark urine, pale stools, and hepatomegaly. Laboratory evaluation revealed elevated AST, ALT, and direct bilirubin levels with normal GGT. Hepatobiliary scintigraphy excluded biliary atresia. UDCA treatment was initiated, and the patient was followed with a preliminary diagnosis of neonatal cholestatic hepatitis of unknown etiology. One year later, jaundice was resolved, laboratory parameters normalized and UDCA treatment was discontinued. A liver biopsy performed at 2 years of age was reported as showing mild chronic hepatitis with stage 3 fibrosis. During follow-up, transaminase and direct bilirubin levels increased again. A subsequent liver biopsy showed chronic hepatitis with periportal bridging fibrosis. An IEBAS was suspected on the basis of persistently normal GGT despite elevated direct bilirubin, parental consanguinity, and the previously observed favorable response to UDCA therapy. The UDCA regimen was recorded as 10 mg/kg twice daily (Table 2). UDCA treatment was discontinued 2 weeks prior to sample collection.

Patient 3: The 9-year-old boy experienced a seizure at 3.5 years of age and had subsequently been followed for developmental delay and loss of previously acquired skills. Tendon xanthomas were noted on clinical examination, and the overall clinical presentation was considered consistent with CTX. Routine biochemical parameters were within reference ranges; however, plasma cholestanol concentration measured in this study was 7.95 µg/mL. Chenodeoxycholic acid (CDCA) treatment (10 mg/kg/day) was started based on clinical suspicion of CTX before the results of genetic analysis became available. CDCA treatment was suspended for the two weeks prior to urine sample collection and subsequently resumed. DNA sequencing of exons 1–9 of the CYP27A1 gene revealed c.508_509ins16, p.(Glu170ValfsTer16) and a deletion mutation. The patient was diagnosed as compound heterozygous for CTX.

Patients 4 and 5: Two sisters, aged 14 and 17 years, were born to consanguineous parents. Both had learning difficulties at school and had undergone bilateral cataract surgery at 8 and 14 years of age, respectively. Juvenile cataract is a characteristic clinical feature of CTX [13]. Routine biochemical parameters were within reference ranges; elevated plasma cholestanol had previously been reported by the referring center. CDCA treatment (10 mg/kg/day) was started based on clinical suspicion of CTX before the results of genetic analysis became available. CDCA treatment was suspended for the two weeks prior to urine sample collection and subsequently resumed. Genetic analysis revealed that both patients were homozygous for the c.646G>C (p.(Ala216Pro), rs201346271) variant, leading to a diagnosis of CTX.

Patient 6: No underlying etiological cause could be established in this patient, who presented with progressive cholestatic hepatitis with normal GGT levels. UDCA treatment (15 mg/kg/day) was initiated. UDCA treatment was discontinued 2 weeks prior to sample collection (Table 2).

Patient 7: The patient was admitted to our hospital at 7 months of age with jaundice. Laboratory evaluation showed elevated direct bilirubin, AST, ALT and INR with normal GGT levels. There was no history of parental consanguinity. UDCA treatment (10 mg/kg twice daily) was initiated. UDCA treatment was discontinued 2 weeks prior to sample collection (Table 2). The patient was subsequently lost to follow-up, and the etiology of the jaundice remained undetermined.

Patient 8: The patient was admitted to our hospital at 2 months of age with jaundice and acholic stools. ALT, AST, direct and total bilirubin levels were elevated, as shown in Table 1. The UDCA (10 mg/kg twice daily) regimen was initiated (Table 2). A Kasai portoenterostomy was performed following a diagnosis of biliary atresia.

Patient 9: The 8-month-old patient presented with direct hyperbilirubinemia and was diagnosed with progressive familial intrahepatic cholestasis type 1 (PFIC1) during follow-up. Recorded UDCA regimens were 30 mg/kg/day and, subsequently, 22 mg/kg/day (Table 2).

Patients 10–12: Clinical and laboratory investigations showed progressive cholestatic hepatitis with normal or mildly elevated GGT levels. No definitive etiological diagnosis was established. The recorded UDCA regimens were 7.5 mg/kg twice daily for patients 10 and 12, and 10 mg/kg twice daily for patient 11 (Table 2).

The demographic characteristics and biochemical data of the patient and control groups are summarized in Table 1.

Table 2 summarizes bile acid treatment in relation to urine collection, including the compound administered, daily dose, treatment interruption interval before sampling, and treatment status at the time of sampling.

### 2.2. Genetic Analyses

Molecular genetic testing included analysis of HSD3B7 in patients 1 and 2 and CYP27A1 in patients 3–5. HSD3B7 sequence variants were described according to the reference transcript NM_025193.4. For patients 1 and 2, genomic DNA was analyzed using a targeted next-generation sequencing panel comprising AMACR, AKR1D1, CYP27A1, and HSD3B7. Sequencing was performed on an Illumina MiSeq platform (San Diego, CA, USA) and covered the coding exons and exon–intron boundaries of the analyzed genes. Identified sequence variants were interpreted in accordance with the American College of Medical Genetics and Genomics (ACMG) criteria.

For patients 3–5, genomic DNA isolated from peripheral blood was analyzed. The coding exons 1–9 of CYP27A1 were amplified by PCR and subjected to DNA sequencing. The results specified the genomic reference sequence NG_007959.1 and the CYP27A1 transcript NM_000784; a transcript version number was not provided. In patient 3, copy-number analysis of CYP27A1 was additionally performed by multiplex ligation-dependent probe amplification (MLPA; assay designation reported as MRCH P300/EK1). Variant interpretation referenced the Human Gene Mutation Database (HGMD) and, for patient 3, MutationTaster. Formal ACMG/AMP pathogenicity categories were not provided. The molecular genetic findings are presented in Section 3, and the diagnostic status of each patient is summarized in Table 1.

### 2.3. Materials

Commercially available reference bile acids (Table A1): GCA, TCA, CA, GCDCA, TCDCA, CDCA, GUDCA, TUDCA, UDCA, GDCA, TDCA, DCA, TLCA and LCA were purchased from Toronto Research Chemicals (Toronto, ON, Canada). GHCA, HCA and GLCA, deuterium standards CA-d_4_, GCA-d_4_, TCA-d_4_, GCDCA-d_4_, TCDCA-d_4_, CDCA-3S-d_5_, GCDCA-3S-d_5_, TCDCA-3S-d_5_ were obtained from Steraloids Inc. (Newport, RI, USA) and Cambridge Isotope Laboratories (Tewksbury, MA, USA), respectively. Commercially unavailable bile acid types (Appendix A) TCA-3S, CA-3S, GCDCA-3S, TCDCA-3S, CDCA-3S, GUDCA-3S, TUDCA-3S, UDCA-3S, GDCA-3S, TDCA-3S, DCA-3S, GLCA-3S, TLCA-3S, LCA-3S, GCA-1β-ol, TCA-1β-ol, CA-1β-ol, GCA-6α-ol, CA-6α-ol, CDCA-1β-ol, G-Δ^5^-3β,7α,12α-(OH)_3_, T-Δ^5^-3β,7α,12α-(OH)_3_, Δ^5^-3β,7α,12α-(OH)_3_, T-Δ^5^-3β,7α,12α-(OH)_3_-3S, Δ^5^-3β,7α,12α-(OH)_3_-3S, G-Δ^5^-3β,7α-(OH)_2_, T-Δ^5^-3β,7α-(OH)_2_, Δ^5^-3β,7α-(OH)_2_, G-Δ^5^-3β,7α-(OH)_2_-3S, T-Δ^5^-3β,7α-(OH)_2_-3S, Δ^5^-3β,7α-(OH)_2_-3S, G-Δ^5^-3β-OH, T-Δ^5^-3β-OH, Δ^5^-3β-OH, G-Δ^5^-3β-OH-3S, T-Δ^5^-3β-OH-3S, Δ^5^-3β-OH-3S, Δ^5^-3β,12α-(OH)_2_, GCA-Δ^4^-3-one, TCA-Δ^4^-3-one, CA-Δ^4^-3-one, GCDCA-Δ^4^-3-one, TCDCA-Δ^4^-3-one, CDCA-Δ^4^-3-one, CA-Δ^4^-6-3-one, CDCA-Δ^4^-6-3-one, nor-CA, C_27_-DHCA, C_27_-THCA were specially synthesized by Prof. Tsuyoshi Murai (Faculty of Pharmaceutical Sciences, Health Sciences University of Hokkaido, Hokkaido, Japan) and Prof. Takashi Iida (Nihon University College of Humanities and Sciences, Department of Chemistry, Tokyo, Japan) and were kindly provided by Hiroshi Nittono (Junshin Clinic Bile Acid Institute, Tokyo, Japan). 7-dehydrocholesterol, cholestanol (5α-cholestan-3β-ol or dihydrocholesterol), cholesterol (Cholest-5-en-3β-ol) and Coprostanol (5β-cholestan-3β-ol) standards for GC–MS analysis were obtained from Sigma Chemical Co. (St. Louis, MO, USA).

### 2.4. LC–MS/MS Analysis

A mixed standard solution containing 66 bile acid species was prepared at 10,000 pmol/mL in 20% acetonitrile. Five-point calibration standards were prepared at 50, 100, 200, 500, 1000 pmol/mL. The stable isotope-labeled internal standards CA-d_4_, GCA-d_4_, TCA-d_4_, GCDCA-d_4_, TCDCA-d_4_, CDCA-3S-d_5_, GCDCA-3S-d_5_, and TCDCA-3S-d_5_ were used for normalization. The mixed internal-standard working solution contained each internal standard at 1000 pmol/mL. For sample extraction, 50 µL of the internal-standard working solution was added to the ethanol extraction solvent before the 2-h incubation. Internal standards were therefore introduced during extraction and were not applied directly to the filter paper with the urine sample. The internal standard assigned to each of the 66 analytes is provided in Table A1. Calibration curves were constructed using the peak-area ratio of each analyte to its assigned internal standard and fitted by unweighted linear regression.

Urine samples were stored at −80 °C until analysis. Dried urine spots were prepared from the stored urine immediately before analysis and were not stored in dried form. For each analysis, 500 µL of urine was applied to a 40-mm-diameter circle on Advantec No. 63 filter paper (Advantec MFS, Dublin, CA, USA) and allowed to dry for 4–5 h at room temperature in a climate-controlled laboratory. The spots were protected from light during drying and relative humidity was not recorded. Each dried urine spot was divided into four equal sections, and one quarter was transferred to a screw-cap vial containing 3 mL of 50% ethanol supplemented with deuterium-labeled internal standards. Samples were incubated at 25 °C for 2 h. After extraction, separate 1-mL aliquots were obtained for bile acid analysis and creatinine measurement. The aliquot used for bile acid analysis was evaporated to dryness, and the residue was reconstituted in 1 mL of sterile water with ultrasonication before LC–MS/MS analysis. Creatinine was measured using a Beckman Coulter AU680 analyzer (Fullerton, CA, USA).

Chromatographic separation was performed using an Inert Sustain C18 column (150 × 2.1 mm i.d., 3 µm; GL Sciences Inc., Tokyo, Japan). Mobile phase A consisted of 10 mM ammonium acetate in water, and mobile phase B consisted of acetonitrile containing 0.01% (*w*/*v*) formic acid. The flow rate was 0.20 mL/min, the column temperature was maintained at 40 °C, and the injection volume was 20 µL. The gradient program was as follows: 0.00–0.50 min, 10% B; 0.50–5.00 min, 10–22% B; 5.00–36.00 min, 22–60% B; 36.00–46.00 min, 60–98% B; 46.00–50.00 min, 98% B; 50.00–50.01 min, 98–10% B; and 50.01–60.00 min, 10% B. The injection volume was 20 µL. The total analytical cycle was therefore 60 min per injection, including re-equilibration at the initial mobile phase composition from 50.01 to 60.00 min, with no additional post-run interval.

The LC system comprised LC-20AD pumps operated in binary-gradient mode and a CTO-10ASvp column oven (Shimadzu Corporation, Kyoto, Japan). The autosampler was maintained at 4 °C. Mass spectrometry analysis was performed using a Shimadzu 8040 triple-quadrupole mass spectrometer (Shimadzu, Kyoto, Japan) equipped with an electrospray ionization source. Classical, hydroxylated, 3β-hydroxy-Δ^5^-, 3-oxo-Δ^4^-, and short- and long-chain bile acids were analyzed in negative-ion mode using multiple reaction monitoring (MRM). The nebulizing and drying gas flow rates were 3 and 15 L/min, respectively. The desolvation line and heat-block temperatures were 250 and 400 °C, respectively. No interface temperature was specified in the analytical method. The interface voltage was 3.5 kV, collision-induced dissociation gas pressure was 230 kPa, detector voltage was 2.30 kV, and conversion-dynode voltage was 6.0 kV. The dwell time was 10 ms per MRM transition. Compound-specific retention times, MRM transitions, collision energies, and coefficients of determination (r^2^) are provided in Table 3 [12,14].

Data acquisition, peak integration, and calibration were performed using Shimadzu LabSolutions LCMS software (version 5.60 SP2). Chromatographic peaks were initially integrated using the software default settings and subsequently inspected visually. No predefined numerical acceptance criteria were applied to peak integration or calibration curves. Calibration standards were analyzed within a single analytical batch, and study samples were analyzed as single injections. No dedicated quality-control samples were included in the LC–MS/MS analytical batch. The injection sequence consisted of calibration standards followed by control and patient samples.

The dried urine spot extraction procedure and LC–MS/MS method were adapted from previously published procedures [12,14]. Analytical evaluation in the present study was limited to the five-point calibration curves and their coefficients of determination reported in Table 3. Urinary bile acid concentrations obtained using this method provide semi-quantitative estimates rather than fully validated quantitative measurements.

### 2.5. GC–MS Analysis

Stock solutions of 7-dehydrocholesterol and 5α-cholestanol were prepared in heptane at concentrations of 624 and 643 µmol/L, respectively, whereas the cholesterol stock solution was prepared in methanol at 1164 µmol/L. Coprostanol (5β-cholestan-3β-ol) was used as an internal standard. Six-point calibration was performed separately for each sterol. The plasma-equivalent calibration concentrations were 11.64, 23.28, 46.56, 93.12, 186.24, and 372.48 mg/dL for cholesterol and 0.3, 0.6, 1.2, 2.4, 4.8, and 9.6 µg/mL for both 7-dehydrocholesterol and 5α-cholestanol. Calibrators were subjected to the same hydrolysis, extraction, evaporation, and derivatization procedures as the study samples. For sample preparation, 100 µL of plasma and 100 µL of coprostanol internal standard solution (0.5 mg/mL in methanol; 50 µg per vial) were added to 3 mL of 0.32 M potassium hydroxide in ethanol. Samples were incubated at 55 °C for 120 min for alkaline hydrolysis. After cooling, 3 mL of water and 2 mL of heptane were added, and the samples were mixed for 10 min. The organic phase was transferred to a glass vial and evaporated to dryness under a stream of nitrogen. The dried extract was derivatized with 50 µL pyridine and 100 µL bis(trimethylsilyl) trifluoroacetamide containing +10% trimethylchlorosilane. Samples were incubated at 80 °C for 1 h before GC–MS. Sterol analysis was performed using a Shimadzu QP2010 GC–MS system (Shimadzu, Kyoto, Japan) operated in electron ionization mode and equipped with an Rtx-1701 capillary column (30 m × 0.25 mm i.d., 0.25 µm film thickness; Restek, Bellefonte, PA, USA). A 1-µL aliquot was injected in split mode at a split ratio of 50:1, with the injector maintained at 300 °C. Helium was used as the carrier gas under linear-velocity control. The oven temperature was maintained at 90 °C for 3 min, increased at 25 °C/min to 260 °C and held for 28 min, then increased at 1 °C/min to 275 °C and held for 13 min. The total run time was 65.8 min. The column head pressure was 49.2 kPa, with a total flow of 43.8 mL/min, a column flow of 0.78 mL/min, a linear velocity of 32.7 cm/s, and a purge flow of 4.0 mL/min. The ion-source and interface temperatures were 200 and 230 °C, respectively. Data were acquired in selected-ion monitoring (SIM) mode. The monitored ions were *m*/*z* 306.4 for 5α-cholestanol, *m*/*z* 325.4 for 7-dehydrocholesterol, and *m*/*z* 329.4 for cholesterol [15]. GC–MS data acquisition and processing were performed using Shimadzu GCMSsolution software (version 4.44). For both calibrators and study samples, coprostanol was added before alkaline hydrolysis and extraction. The analytical response for each sterol was expressed as the analyte-to-coprostanol peak-area ratio multiplied by 100. Separate calibration curves were constructed for cholesterol, 7-dehydrocholesterol, and 5α-cholestanol. Study-specific validation data for accuracy, intra- and inter-assay precision, selectivity, extraction recovery, limits of quantification, and analyte stability were not available.

### 2.6. Statistical Analysis

Statistical analyses were performed using IBM SPSS Statistics for Windows (version 22.0; IBM Corp., Armonk, NY, USA). Analyses were descriptive because of the clinical heterogeneity of the cohort and the small number of patients within individual diagnostic categories; therefore, no inferential comparisons between patients and controls were performed. Control age, clinical biochemical variables, and plasma sterol concentrations are presented as mean ± standard deviation, whereas individual patient values are reported separately. Categorical variables are summarized as counts and, where appropriate, percentages. Table 4 presents urinary bile acid concentrations and their relative contributions to total urinary bile acids. For each individual, the percentage contribution of a bile acid group was calculated as the concentration of that group divided by total urinary bile acid concentration × 100. Control concentrations and percentages are reported as arithmetic means calculated among individuals with an available positive numerical result for the corresponding bile acid group. Because the number of contributing control samples varied among bile acid groups, the mean percentages across groups are not expected to sum to 100%. In Supplementary Table S1, bile acid concentrations in controls and patients are summarized as arithmetic mean (minimum–maximum) together with the frequency of available positive numerical results, expressed as *n*/*N* (%), where *N* = 50 for controls and *N* = 12 for patients. When *n* = 1, the single observed value is reported without a range; when *n* = 0, the concentration is indicated as unavailable and the frequency as 0/*N* (0%). Zero-coded entries and observations without a numerical result were excluded from summary calculations, without imputation. Zero-coded entries were not interpreted as analytically confirmed zero concentrations. Group totals were calculated as the sum of available numerical results for the constituent bile acids; when none of the constituent analytes had an available numerical result, the group total was considered unavailable. Analyte-specific validated limits of detection (LOD) and lower limits of quantification (LLOQ) were not established in this study. Consequently, the dataset does not permit distinction between the true absence of an analyte and a signal below the analytical detection or quantification limit. Detection frequencies therefore represent the frequency of recorded positive numerical results rather than analytically validated detection rates. Minimum–maximum values represent the observed range among participants with available positive numerical results and were used only for descriptive comparison of individual patient profiles. These ranges were not interpreted as population-based reference intervals or diagnostic cutoffs. Because percentage contributions are compositional variables sharing total urinary bile acids as a common denominator, they were interpreted together with the corresponding absolute concentrations. In particular, a low absolute concentration may constitute a relatively large percentage when the total urinary bile acid concentration is low. Conversely, marked accumulation of a treatment-related or otherwise dominant bile acid species may reduce the proportional contribution of other components without a corresponding reduction in their absolute concentrations. Additional descriptive analyses were performed to explore selected biochemical patterns. The 3β-hydroxy-Δ^5^/classical bile acid concentration ratio was calculated for patients 1 and 2, and the 3-oxo-Δ^4^/classical bile acid concentration ratio for patients 3–5. Ratios were calculated only when both group totals were available, and the denominator was greater than zero. These ratios are dimensionless descriptive measures and were not interpreted using validated diagnostic thresholds. In an exploratory analysis, analytes assigned as UDCA, GUDCA, TUDCA, and their 3-sulfate conjugates were excluded from patient and control profiles. Group concentrations and their percentage contributions to the retained total urinary bile acid concentration were then recalculated and are presented in the second block of Table 4. Patient group totals were derived from the individual-species concentrations reported in Supplementary Table S1. For controls, recalculation was performed at the individual-participant level before group averaging, using the same inclusion rules as in the primary analysis. This exploratory omission was based on the reported analyte assignments and should not be interpreted as complete removal of all UDCA-derived metabolites or as a formal correction for treatment effects. For patients 1 and 3–5, the plasma cholestanol-to-cholesterol mass ratio was calculated after converting cholesterol concentrations from mg/dL to µg/mL. Cholestanol concentration (µg/mL) was then divided by cholesterol concentration (µg/mL).

### 2.7. Use of Generative Artificial Intelligence

**Figure 1:**
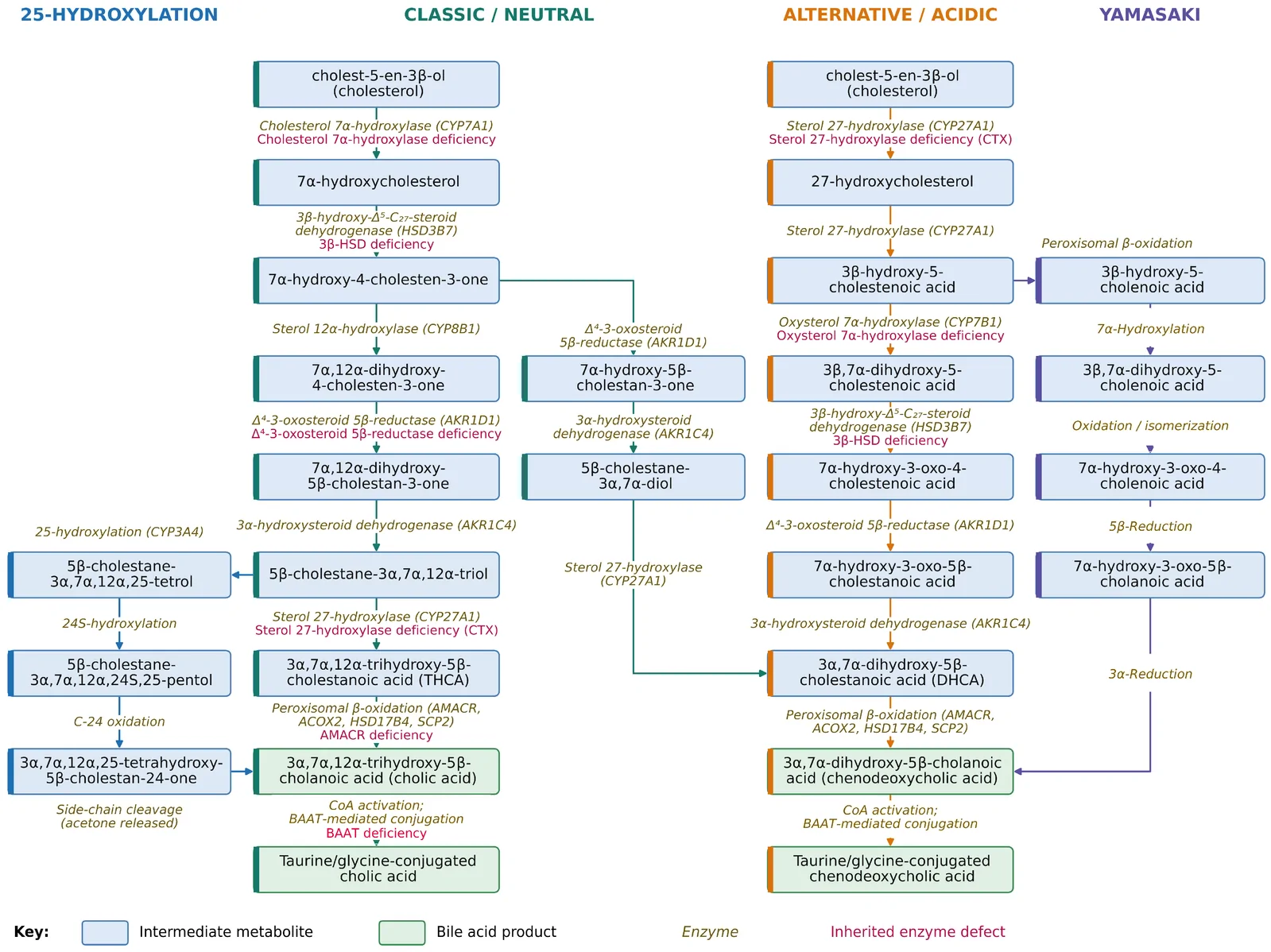
Bile acid synthesis pathways. Schematic representation of the classical/neutral and alternative/acidic pathways [6], together with the 25-hydroxylation and Yamasaki routes. Blue boxes represent intermediate metabolites, and green boxes indicate bile acid products. Arrows show the direction of the depicted conversions; enzyme names or reaction types are indicated alongside the arrows. Red labels identify inherited enzyme deficiencies. CTX, cerebrotendinous xanthomatosis; 3β-HSD, 3β-hydroxy-Δ^5^-C_27_-steroid dehydrogenase/isomerase; BAAT, bile acid CoA–amino acid N-acyltransferase; THCA, trihydroxycholestanoic acid; DHCA, dihydroxycholestanoic acid.

OpenAI ChatGPT (GPT-6) was used for language editing, manuscript formatting, and graphical refinement of Figure 1. The authors reviewed and verified the scientific accuracy of all outputs and take full responsibility for the final content.

## 3. Results

Five patients had molecularly confirmed inborn errors of bile acid synthesis: HSD3B7 deficiency in patients 1 and 2 and cerebrotendinous xanthomatosis (CTX) in patients 3–5. Patient 8 had biliary atresia, and patient 9 had progressive familial intrahepatic cholestasis type 1. No definitive etiological diagnosis was established in patients 6, 7, or 10–12. The enzyme deficiencies considered in patients 6 and 7 were based on biochemical findings and remained unconfirmed. Molecular genetic analysis identified homozygous HSD3B7 variants in patients 1 and 2: NM_025193.4:c.598C>T (p.(Gln200Ter)) and NM_025193.4:c.45_46del (p.(Gly17LeufsTer26)), respectively. According to the respective laboratory reports, the variant identified in patient 1 was classified as likely pathogenic and that identified in patient 2 as pathogenic according to ACMG criteria. Patient 3 was heterozygous for a CYP27A1 insertion reported as c.508_509ins16 (p.(Glu170ValfsTer16)). Subsequent multiplex ligation-dependent probe amplification (MLPA) demonstrated a heterozygous single-copy loss of exon 6, reported as “rsa 2q35 (CYP27A1 exon 6)x1.” The father was heterozygous for p.(Glu170ValfsTer16), whereas the mother was reported to be homozygous for the CYP27A1 exon 6 deletion. These parental findings support the presence of the two CYP27A1 variants in trans in patient 3 and are consistent with compound heterozygosity. Patients 4 and 5 were homozygous for the CYP27A1 variant c.646G>C (p.(Ala216Pro); rs201346271). This variant was identified as disease-causing for CTX based on information available in the Human Gene Mutation Database (HGMD).

Results for the 66 urinary bile acid species analyzed by LC–MS/MS are reported as semi-quantitative estimates normalized to urinary creatinine (µmol/mmol creatinine). Calibration curves covered the range of 50–1000 pmol/mL, and the corresponding coefficients of determination (r^2^) are presented in Table 3. Bile acid species were classified into five groups: classical, hydroxylated, 3β-hydroxy-Δ^5^-, 3-oxo-Δ^4^-, and short- and long-chain bile acids. For each profile, both group concentrations and their percentage contributions to total urinary bile acids were evaluated. Plasma cholesterol measured by GC–MS is reported in mg/dL, whereas 5α-cholestanol and 7-dehydrocholesterol are reported in µg/mL (Table 4). Individual urinary bile acid results for patients and controls are provided in Supplementary Table S1.

In patient 1, the total concentration of 3β-hydroxy-Δ^5^ bile acids was 21.011 µmol/mmol Cr, corresponding to 63.9% of total urinary bile acids. The predominant species included Δ^5^-3β,7α-(OH)_2_-3S (14.549 µmol/mmol Cr), G-Δ^5^-3β,7α-(OH)_2_ (4.394 µmol/mmol Cr), and Δ^5^-3β,7α,12α-(OH)_3_ (1.816 µmol/mmol Cr) (Table 4 and Supplementary Table S1).

In patient 2, the total 3β-hydroxy-Δ^5^ bile acid concentration was 0.158 µmol/mmol Cr, corresponding to 22.1% of total urinary bile acids. Δ^5^-3β-OH was present at 0.141 µmol/mmol Cr. Total urinary bile acids were 0.72 µmol/mmol Cr, which fell within the observed range among controls. The 3β-hydroxy-Δ^5^/classical bile acid concentration ratios were 1.799 in patient 1 and 0.301 in patient 2. In patient 2, Δ^5^-3β,7α-(OH)_2_-3S was reported at 0.001 µmol/mmol Cr, whereas no numerical results were recorded for G-Δ^5^-3β,7α-(OH)_2_-3S or Δ^5^-3β,7α,12α-(OH)_3_-3S (Supplementary Table S1). Thus, patient 2 did not show the marked predominance of sulfated dihydroxy-Δ^5^ bile acids observed in patient 1, representing an atypical urinary biochemical profile in the context of the molecularly confirmed HSD3B7 diagnosis.

Patient 3, who had compound-heterozygous *CYP27A1* variants and a confirmed diagnosis of CTX, had urinary CDCA-1β-ol and CA-Δ^4^,6-3-one concentrations of 0.128 and 0.163 µmol/mmol Cr, respectively (Supplementary Table S1). The total hydroxylated and 3-oxo-Δ^4^ bile acid concentrations were 0.133 and 0.170 µmol/mmol Cr, corresponding to 19.7% and 25.1% of total urinary bile acids, respectively. Classical bile acids accounted for 50.7% of the urinary bile acid profile. Total urinary bile acids were 0.68 µmol/mmol Cr, within the observed control range (Table 4). The 3-oxo-Δ^4^/classical bile acid ratio was 0.496. The 3β-hydroxy-Δ^5^ bile acid concentration was 0.031 µmol/mmol Cr, also within the observed control range.

In siblings 4 and 5, with confirmed CTX, CA-Δ^4^,6-3-one concentrations were 0.307 and 0.214 µmol/mmol Cr, respectively, whereas G-Δ^5^-3β-OH-3S concentrations were 0.069 and 0.045 µmol/mmol Cr (Supplementary Table S1). Both G-Δ^5^-3β-OH-3S concentrations were within the observed control range (0.002–0.122 µmol/mmol Cr), with positive numerical results reported in 38 of 50 controls (Supplementary Table S1). Patient 4 additionally had TCA-1β-ol at 0.149 µmol/mmol Cr. In patient 4, the 3-oxo-Δ^4^, 3β-hydroxy-Δ^5^, and hydroxylated bile acid groups accounted for 36.2%, 30.2%, and 17.1% of total urinary bile acids, respectively. In patient 5, the 3-oxo-Δ^4^ and 3β-hydroxy-Δ^5^ groups accounted for 21.6% and 13.6%, respectively; no numerical result was reported for the hydroxylated bile acid group. Total urinary bile acid concentrations were 0.99 and 1.14 µmol/mmol Cr in patients 4 and 5, respectively, both within the observed control range. The corresponding 3-oxo-Δ^4^ group concentrations were 0.357 and 0.247 µmol/mmol Cr, and the 3β-hydroxy-Δ^5^ group concentrations were 0.298 and 0.156 µmol/mmol Cr. The 3-oxo-Δ^4^/classical bile acid ratios were 2.204 and 0.334, respectively (Table 4). The plasma cholestanol/cholesterol mass ratios were 3.11 × 10^−3^, 2.58 × 10^−3^, and 2.01 × 10^−3^ in patients 3, 4, and 5, respectively. For descriptive comparison, the corresponding ratio in patient 1 with HSD3B7 deficiency was 2.57 × 10^−3^. These ratios were calculated from the individual sterol concentrations reported in Table 4. In controls, the plasma cholestanol/cholesterol mass ratio was (1.28 ± 0.33) × 10^−3^ (mean ± SD; *n* = 46), calculated from individual paired concentrations after conversion to the same mass concentration units.

In patient 6, urinary TCA-1β-ol and CA-Δ^4^,6-3-one concentrations were 1.45 and 0.437 µmol/mmol Cr, respectively (Supplementary Table S1). Hydroxylated and 3-oxo-Δ^4^ bile acids accounted for 47.5% and 13.4% of total urinary bile acids, respectively, whereas classical bile acids accounted for 38.4% (Table 4).

In patient 7, Δ^5^-3β-OH was detected at 1.346 µmol/mmol Cr. The 3β-hydroxy-Δ^5^ and classical bile acid groups accounted for 18.1% and 68.9% of total urinary bile acids, respectively (Table 4 and Supplementary Table S1). C_27_-DHCA and C_27_-THCA were also reported at concentrations of 0.059 and 0.073 µmol/mmol Cr, respectively.

Patients 8–12 were receiving UDCA at the time of urine collection (Table 2). GUDCA was the predominant assigned species in patients 8–11, and classical bile acids comprised the largest proportion of their urinary profiles. Patient 8 also had detectable CA-Δ^4^,6-3-one and GCA-6α-ol, whereas GCA-6α-ol was additionally detected in patient 11. CA-Δ^4^,6-3-one was detected in patients 9 and 10. Patient 12 had a lower GUDCA concentration than patients 8–11, although GUDCA remained the predominant assigned species; T-Δ^5^-3β-OH-3S and CA-Δ^4^,6-3-one were also detected. Group concentrations and percentage contributions are presented in Table 4, and individual bile acid species are detailed in Supplementary Table S1.

In the exploratory analysis excluding species assigned as UDCA, GUDCA, TUDCA, and their 3-sulfate conjugates, the retained classical bile acid concentrations in patients 8–12 were 1.091, 31.614, 15.866, 7.594, and 0.380 µmol/mmol Cr, respectively (second block of Table 4). In patient 8, the 3-oxo-Δ^4^ group was the largest retained group at 1.276 µmol/mmol Cr, of which CA-Δ^4^,6-3-one accounted for 0.904 µmol/mmol Cr. Classical bile acids remained the largest retained group in patients 9–11. In patient 12, the retained classical, 3-oxo-Δ^4^, and 3β-hydroxy-Δ^5^ bile acid concentrations were 0.380, 0.220, and 0.161 µmol/mmol of Cr, respectively.

## 4. Discussion

Inborn errors of bile acid synthesis (IEBAS) encompass a heterogeneous group of disorders with clinical manifestations ranging from neonatal or infantile cholestatic liver disease to later-onset neurological involvement. Importantly, several of these disorders are amenable to specific treatment, making timely recognition clinically relevant. In this descriptive case series, urinary profiles of 66 bile acid species were characterized in 12 patients evaluated for suspected IEBAS or unexplained cholestasis and compared descriptively with those of 50 healthy controls. Five patients had genetically confirmed IEBAS: HSD3B7 deficiency in patients 1 and 2 and cerebrotendinous xanthomatosis (CTX) in patients 3–5. Patient 8 had biliary atresia and patient 9 had progressive familial intrahepatic cholestasis type 1 (PFIC1), whereas no definitive etiological diagnosis was established in patients 6, 7, and 10–12. The findings therefore illustrate the range of urinary bile acid patterns encountered during the diagnostic evaluation of patients with confirmed, suspected, or alternative causes of cholestatic disease.

### 4.1. 3β-Hydroxy-Δ^5^-Steroid Dehydrogenase Deficiency (HSD3B7)

In patient 1 with genetically confirmed HSD3B7 deficiency, the urinary bile acid profile was characterized by prominent 3β-hydroxy-Δ^5^ metabolites, including sulfated dihydroxy-Δ^5^, glycine-conjugated dihydroxy-Δ^5^, and trihydroxy-Δ^5^ bile acids. Such metabolites are well-established biochemical features of 3β-hydroxy-Δ^5^-C_27_-steroid dehydrogenase/isomerase deficiency [9,11].

The HSD3B7 gene encodes 3β-hydroxy-Δ^5^-C_27_-steroid dehydrogenase/isomerase, which catalyzes oxidation of the 3β-hydroxyl group of 7α-hydroxycholesterol to a 3-oxo group together with isomerization of the Δ^5^ double bond to Δ^4^, yielding 7α-hydroxy-4-cholesten-3-one [16]. Subsequent reactions involving Δ^4^-3-oxosteroid 5β-reductase and 3α-hydroxysteroid dehydrogenase establish the 3α-hydroxy-5β configuration characteristic of primary bile acids. Loss of HSD3B7 activity disrupts this early steroid-ring modification step and results in accumulation of bile acid intermediates retaining the 3β-hydroxy-Δ^5^ configuration, including 3β,7α-dihydroxy-5-cholenoic acid and 3β,7α,12α-trihydroxy-5-cholenoic acid [9]. These C_24_ bile acids may subsequently undergo sulfation at the 3β-hydroxyl group and glycine or taurine conjugation at the side-chain carboxyl group [17].

Accordingly, patients with HSD3B7 deficiency typically show reduced formation of conventional primary bile acids together with urinary accumulation of sulfated and conjugated 3β-hydroxy-Δ^5^ intermediates [9,18]. In patient 1, the total 3β-hydroxy-Δ^5^ bile acid concentration was 21.011 µmol/mmol Cr and constituted the predominant abnormal metabolite group. The presence and distribution of the individual sulfated dihydroxy-, glycine-conjugated dihydroxy-, and trihydroxy-Δ^5^ species were consistent with the expected biochemical phenotype and provided metabolic characterization complementary to the molecular diagnosis.

The concentrations assigned to GDCA-3S and TDCA-3S in patient 1 (3.62 and 4.84 µmol/mmol Cr, respectively; Supplementary Table S1) should, however, be interpreted cautiously because of possible analytical interference from structurally related isomers. For this reason, interpretation was based primarily on the genetically established diagnosis, the overall 3β-hydroxy-Δ^5^ bile acid pattern, the absolute concentration of this metabolite group, and the distribution of individual metabolites within the group rather than on any single isomeric species.

In patient 2, the 3β-hydroxy-Δ^5^ bile acid concentration was 0.158 µmol/mmol Cr, accounting for 22.1% of a total urinary bile acid concentration of 0.715 µmol/mmol Cr. This concentration was slightly higher than the highest observed value among controls with available numerical results (0.128 µmol/mmol Cr), although the control range was descriptive and was not established as a reference interval. In contrast to patient 1, however, patient 2 showed an atypical biochemical phenotype, with the monohydroxy species Δ^5^-3β-OH predominating within the 3β-hydroxy-Δ^5^ fraction. The diagnosis of HSD3B7 deficiency was established by the identification of a homozygous pathogenic HSD3B7 variant.

Predominance of 3β-monohydroxy-Δ^5^-C_24_ bile acids is not specific to a single bile acid synthesis defect. Kimura et al. reported excessive urinary 3β-monohydroxy-Δ^5^-C_24_ bile acids in HSD3B7 deficiency and emphasized that this pattern does not necessarily indicate primary oxysterol 7α-hydroxylase deficiency [19]. Similarly, Mizuochi et al. described substantial urinary Δ^5^-3β-OH excretion in a patient with HSD3B7 deficiency [20]. These observations support the possibility of biochemical heterogeneity within genetically confirmed HSD3B7 deficiency and underscore the importance of interpreting urinary bile acid profiles together with molecular findings rather than assigning a diagnosis on the basis of a single metabolite species.

The classical bile acid concentration in patient 2 was 0.525 µmol/mmol Cr, accounting for 73.4% of total urinary bile acids. The signal assigned to GUDCA contributed 0.433 µmol/mmol Cr, corresponding to 82.5% of the classical fraction and 60.6% of total urinary bile acids (Table 4 and Supplementary Table S1). However, chromatographic separation of GUDCA from GCDCA and GDCA was not verified; therefore, contributions from incompletely resolved isomers cannot be excluded. Although UDCA treatment had been discontinued 2 weeks before sampling, this signal cannot be confidently attributed to residual treatment. After omission of UDCA-assigned species, the retained classical bile acid concentration was approximately 0.09 µmol/mmol Cr, representing 32.4% of the retained total, whereas the 3β-hydroxy-Δ^5^ fraction increased from 22.1% to 56.2%. The absolute concentration of 3β-hydroxy-Δ^5^ bile acids remained unchanged at 0.158 µmol/mmol Cr. This percentage increase reflects the reduced denominator and, given the uncertainty in the excluded analyte assignments, does not independently establish a characteristic HSD3B7 biochemical profile.

### 4.2. Sterol 27-Hydroxylase Deficiency [Cerebrotendinous Xanthomatosis (CTX)]

Patients 3–5 had genetically confirmed CTX but showed heterogeneous urinary bile acid profiles. Hydroxylated bile acids were detected in patients 3 and 4, whereas no numerical result was available for this group in patient 5. CA-Δ^4^,6-3-one was detected in all 12 patients and reached its highest concentration in patient 8 with biliary atresia; therefore, the presence of this metabolite was not specific to CTX in the present series.

Sterol 27-hydroxylase is a mitochondrial enzyme encoded by CYP27A1 and participates in both the classical/neutral and alternative/acidic pathways of bile acid synthesis by catalyzing sterol 27-hydroxylation (Figure 1) [21]. Its deficiency causes CTX, an autosomal recessive disorder characterized by progressive neurological dysfunction, juvenile cataracts, tendon xanthomas, and other systemic manifestations [22,23,24]. In CTX, impairment of CYP27A1 activity reduces primary bile acid synthesis and promotes accumulation of sterol intermediates, cholestanol, and bile alcohols [7]. Bile alcohol glucuronides represent an important biochemical feature of CTX [25], although these metabolites were not included in the analytical panel used in the present study.

Plasma cholestanol concentrations were 7.95, 4.16, and 3.44 µg/mL in patients 3–5, respectively, compared with a control mean of 2.68 ± 0.99 µg/mL. However, patient 1 with HSD3B7 deficiency had a plasma cholestanol concentration of 5.43 µg/mL, exceeding the concentrations observed in patients 4 and 5 (Table 4). Thus, cholestanol concentration alone did not discriminate CTX from other disorders in this small cohort. This is consistent with previous observations that increased plasma cholestanol is not specific for CTX and may occur in other liver diseases [26]. The diagnoses of CTX in patients 3–5 were therefore based primarily on their clinical phenotypes and CYP27A1 findings, with plasma sterol and urinary bile acid measurements providing complementary biochemical characterization.

Patient 6 showed a different clinical situation. Classical, hydroxylated, and 3-oxo-Δ^4^ bile acids accounted for 38.4%, 47.5%, and 13.4% of total urinary bile acids, respectively (Table 4). Although this distribution raised CTX among the biochemical differential considerations, the patient remained without a definitive etiological diagnosis. No plasma sample was available for cholestanol measurement, and molecular confirmation was not obtained. In addition, patient 6 was only 4 months old; therefore, the urinary bile acid profile must be interpreted in the context of developmental variation associated with infancy as well as the underlying cholestatic liver disease.

### 4.3. Biochemical Hypotheses in Patient 7 with Unresolved Cholestasis

The urinary bile acid profile of patient 7 prompted consideration of defects affecting early steps of bile acid synthesis, including CYP7B1, CYP7A1, and HSD3B7, although no definitive etiological diagnosis was established. Δ^5^-3β-OH was the predominant component of the 3β-hydroxy-Δ^5^ bile acid group, with a concentration of 1.346 µmol/mmol Cr, whereas the total 3β-hydroxy-Δ^5^ concentration was 1.781 µmol/mmol Cr. C_27_-DHCA and C_27_-THCA were also detected, raising peroxisomal disorders as an additional biochemical consideration because cholestanoic acid intermediates may accumulate in disorders of peroxisomal bile acid metabolism [11].

Oxysterol 7α-hydroxylase, encoded by CYP7B1, catalyzes 7α-hydroxylation of oxysterol intermediates in the alternative/acidic pathway, whereas cholesterol 7α-hydroxylase, encoded by CYP7A1, catalyzes the initial 7α-hydroxylation of cholesterol in the classical/neutral pathway (Figure 1). The relative contribution of these pathways varies with age, with the alternative pathway having greater importance during early life [7,27]. Consequently, interpretation of an unusual monohydroxy-Δ^5^ bile acid profile in a 7-month-old infant requires particular caution.

Although the findings in patient 7 generated biochemical hypotheses, none of the observed metabolites or group distributions were considered diagnostic of a specific enzyme defect. The patient was subsequently lost to follow-up, precluding further molecular or biochemical clarification. This case illustrates the value of broad bile acid profiling for generating differential diagnostic hypotheses, while also demonstrating the limitations of assigning a specific defect in the absence of confirmatory molecular or longitudinal evidence.

### 4.4. Influence of Bile Acid Treatment on Urinary Profiles

Patients 8–12 were receiving UDCA at the time of urine collection (Table 2), and their urinary profiles were dominated by species assigned to UDCA metabolism, particularly GUDCA. The clinical diagnoses in these patients were heterogeneous: patient 8 had biliary atresia, patient 9 had PFIC1, and patients 10–12 remained without a definitive etiological diagnosis. Consequently, the predominance of UDCA-related species was interpreted primarily in the context of documented treatment rather than as a disease-specific metabolic pattern.

Free bile acids and their conjugated derivatives were reported as separate analytes in this study. Notably, no positive numerical results were reported for unconjugated UDCA, CDCA, or DCA in any of the 62 participants, including patients 8–12, who were receiving UDCA at sampling. The species assigned to GUDCA was present in all five UDCA-treated patients and predominated in their measured urinary profiles.

This observation aligns with clinical urinary profiling studies which demonstrate that both endogenous and exogenous bile acids undergo rapid and extensive hepatic and renal biotransformation. Following intestinal absorption and first-pass hepatic extraction, administered UDCA is efficiently converted into glycine- and taurine-conjugated UDCA, as well as its sulfate and N-acetylglucosamine conjugates. Because renal excretion overwhelmingly favors these highly water-soluble conjugated species, unconjugated UDCA, CDCA, and DCA remain at negligible levels or fall below the limit of quantification in urine [28]. These findings support the interpretation that measurement of unconjugated UDCA alone does not capture the full spectrum of urinary UDCA derivatives. The absence of reported positive numerical results for unconjugated UDCA, CDCA, and DCA was therefore considered alongside the findings for conjugated species. Since analyte-specific limits of detection and quantification were not established, this observation was not interpreted as evidence that the unconjugated species were absent from the samples or had undergone complete conversion.

The exploratory analysis excluding species assigned as UDCA, GUDCA, TUDCA, and their 3-sulfate conjugates provided a descriptive view of the remaining measured bile acid components (second block of Table 4). Importantly, this analysis does not reconstruct an untreated metabolic profile, because bile acid therapy may alter endogenous bile acid synthesis, pool composition, and feedback regulation. Similarly, ratios between atypical and classical bile acid groups were interpreted together with their absolute concentrations. For example, the relatively high 3-oxo-Δ^4^/classical ratio in patient 4 occurred in the setting of a low absolute classical bile acid concentration. In controls, a positive numerical result assigned to GUDCA was recorded in all 50 individuals, with a mean concentration of 0.515 µmol/mmol Cr, compared with 0.001 µmol/mmol Cr for GCDCA and 0.008 µmol/mmol Cr for GCA (Supplementary Table S1). Because chromatographic separation of GUDCA, GCDCA, and GDCA was not verified, the apparent predominance of GUDCA cannot be confidently interpreted as a biological finding. Omission of UDCA-assigned species reduced the mean control total from 0.59 to 0.08 µmol/mmol Cr, predominantly through removal of the GUDCA-assigned signal. If this signal includes contributions from other isomers, those contributions would also be excluded. Accordingly, the retained totals and percentages in Table 4 depend on the original analyte assignments and should not be interpreted as a verified non-UDCA bile acid profile.

Treatment status is particularly relevant when urinary bile acid profiling is used during the diagnostic evaluation of suspected IEBAS. Long-term cholic acid treatment has been associated with suppression of atypical urinary bile acid metabolites, with increases reported during periods of poor adherence [29]. UDCA treatment has likewise been shown to alter urinary bile acid composition [30].

The present study was not designed to determine an appropriate diagnostic washout interval, because serial sampling before and after treatment withdrawal was not performed. Moreover, withdrawal of bile acid therapy may carry clinical risks. In the RESTORE trial involving patients with CTX aged ≥16 years, withdrawal of chenodeoxycholic acid resulted in increases in disease biomarkers, and rescue treatment was required in a substantial proportion of participants [31]. Similarly, deterioration in liver biochemistry has been reported following UDCA withdrawal in patients with primary sclerosing cholangitis [32]. Therefore, routine withdrawal of clinically necessary bile acid therapy solely to facilitate urinary profiling cannot be recommended on the basis of the present data. When treatment interruption is considered for a specific diagnostic purpose, the potential benefit should be weighed against the clinical status of the individual patient and undertaken with appropriate monitoring.

### 4.5. Strengths and Limitations

The strength of the present study is the comprehensive targeted assessment of 66 urinary bile acid species encompassing classical, hydroxylated, 3β-hydroxy-Δ^5^-, 3-oxo-Δ^4^, and short- and long-chain bile acids. This broad analytical coverage enabled characterization of individual bile acid species as well as their distribution across metabolite groups and allowed the biochemical findings to be interpreted alongside clinical and molecular data. The inclusion of genetically confirmed HSD3B7 deficiency and CTX cases also provided an opportunity to examine biochemical heterogeneity even within established disorders.

However, certain limitations should nevertheless be considered. First, the study comprised a small and clinically heterogeneous cohort, reflecting the rarity of IEBAS, precluding meaningful disease-specific statistical comparisons. Second, the age difference between patients and controls represents a limitation. Urinary bile acid composition undergoes developmental changes during infancy, including variation in hydroxylated and unsaturated ketonic bile acid species. Urinary creatinine excretion is also influenced by age, body size, and muscle mass; therefore, normalization to creatinine does not eliminate age-related differences. For this reason, profiles from infants were interpreted descriptively in the context of age, cholestasis, and treatment, with the older controls serving as a comparison group rather than as a source of age-specific reference intervals. Third, bile acid therapy may represent a potential confounder. The urinary profiles were assessed cross-sectionally and reported urinary bile acid concentrations should be regarded as semi-quantitative estimates.

Incomplete chromatographic discrimination of certain structurally isomeric bile acids limits unequivocal species-level assignment, including GCDCA-3S/GDCA-3S and GCDCA/GUDCA/GDCA. This uncertainty also affects analyses that selectively omit an affected species. In particular, the predominance of the GUDCA-assigned signal in controls and in the classical fraction of patient 2 limits interpretation of the exploratory analysis excluding UDCA-assigned species. The retained totals, relative group contributions, and comparisons based on these quantities are therefore conditional on the original assignments. The molecularly established diagnoses are independent of this analytical uncertainty, but biochemical interpretations involving the affected signals and derived proportions require caution. This study was designed to characterize biochemical profiles that complement, rather than replace, clinical and molecular evaluation.

## 5. Conclusions

In this descriptive case series, comprehensive urinary bile acid profiling provided biochemical information that complemented clinical and molecular findings in patients with genetically confirmed IEBAS, as well as in patients with other or unresolved causes of cholestasis. These findings support the use of urinary bile acid profiling as a complementary biochemical approach for identifying characteristic metabolic patterns and guiding molecular investigation in patients with suspected bile acid synthesis disorders.

However, this study was not designed to establish diagnostic sensitivity, specificity, or clinically validated decision thresholds. Larger studies including genetically characterized cohorts, appropriate reference populations, and fully validated quantitative analytical methods are needed to define the diagnostic performance of comprehensive urinary bile acid profiling and to determine its clinical utility for treatment assessment and longitudinal monitoring.

## Figures and Tables

**Table 1:** Demographic characteristics and biochemical findings of the patients and controls are summarized in this table.

	Controls, *n* = 50	P1	P2	P3	P4	P5	P6	P7	P8	P9	P10	P11	P12
Diagnostic status		HSD3B7 (A)	HSD3B7 (A)	CTX (A)	CTX (A)	CTX (A)	Unresolved	Unre-solved	Biliary atresia (B)	PFIC1 (B)	Unresolved	Unresolved	Unresolved
Gender	24:26 (F/M)	M	M	M	F	F	F	M	M	F	M	M	F
Age	9.84 ± 4.72	8 y	15 y	9 y	14 y	17 y	4 m	7 m	2 m	8 m	12 y	8 m	1 y
ALT (U/L) (r.r. < 39)	17.68 ± 10.25	**919**	**51**	16	13	14	**250**	**120**	**285**	**67**	**127**	**86**	**83**
AST (U/L) (r.r. < 56)	25.24 ± 8.95	**275**	**56**	24	20	26	**469**	**171**	**447**	**164**	53	**281**	**190**
GGT (U/L) (r.r. 3–22)	n.d.	15	6.1	12	9	6	46 (r.r. 12–122)	60 (r.r. 12–122)	104 (r.r. 12–122)	40 (r.r. 12–122)	**58**	66 (r.r. 12–122)	**29**
D-Bil (mg/dL) (r.r. 0–0.2)	0.11 ± 0.05	**3.92**	**4.64**	0.15	0.10	0.09	**4.0**	**3.60**	**3.91**	**3.11**	**0.33**	**17.98**	**0.28**
T-Bil (mg/dL) (r.r. 0.3–1.2)	0.50 ± 0.22	**6.78**	**6.78**	0.69	0.51	0.40	**8.6**	**7.09**	**7.47**	**5.42**	1.07	**25.07**	1.2

Reference intervals were taken from the clinical laboratory reports. The GGT interval of 12–122 U/L applies to P6, P7, P8, P9, and P11 and is shown in their respective cells; the row-heading interval of 3–22 U/L applies to the other patients. For the other biochemical parameters, the intervals are shown in the row headings. Values above the applicable reference range are indicated in bold. Control-group age and biochemical data are presented as mean ± SD; sex distribution is reported as female/male counts. Individual patient data are shown as observed values. A, genetically confirmed IEBAS (HSD3B7 deficiency in P1 and P2; CTX in P3–P5); B, received another diagnosis. Unresolved indicates that no definitive etiological diagnosis was established. *n*, number of participants; P, patient; F, female; M, male; y, years; m, months; r.r., reference range; ALT, alanine aminotransferase; AST, aspartate aminotransferase; GGT, gamma-glutamyltransferase; D-Bil, direct bilirubin; T-Bil, total bilirubin; CTX, cerebrotendinous xanthomatosis; PFIC1, progressive familial intrahepatic cholestasis type 1; n.d., not determined.

**Table 2:** Bile acid treatment in relation to urine collection.

Patient	Compound	Daily Dose	Treatment Duration	Interruption Interval	Treatment Status at Sampling
1	UDCA	15 mg/kg/day	2 years	2 weeks	UDCA discontinued
2	UDCA	10 mg/kg, twice daily	2 years	2 weeks	UDCA discontinued
3	CDCA	10 mg/kg/day	1 year	2 weeks	CDCA discontinued
4	CDCA	10 mg/kg/day	1 year	2 weeks	CDCA discontinued
5	CDCA	10 mg/kg/day	1 year	2 weeks	CDCA discontinued
6	UDCA	15 mg/kg/day	1 month	2 weeks	UDCA discontinued
7	UDCA	10 mg/kg, twice daily	1 month	2 weeks	UDCA discontinued
8	UDCA	10 mg/kg, twice daily	1 week	Not interrupted	Receiving UDCA
9	UDCA	30 mg/kg/day; subsequently 22 mg/kg/day	3 weeks	Not interrupted	Receiving UDCA
10	UDCA	7.5 mg/kg, twice daily	5 years	Not interrupted	Receiving UDCA
11	UDCA	10 mg/kg, twice daily	4 months	Not interrupted	Receiving UDCA
12	UDCA	7.5 mg/kg, twice daily	1 week	Not interrupted	Receiving UDCA

UDCA, ursodeoxycholic acid; CDCA, chenodeoxycholic acid. Treatment duration represents the approximate duration of treatment for each patient, as determined from the available retrospective clinical records. The interruption interval denotes the time between treatment discontinuation and urine collection.

**Table 3:** Retention times (RT), multiple reaction monitoring (MRM) transitions, collision energies (eV), and coefficients of determination (r^2^) for the bile acid species analyzed by LC–MS/MS.

Bile Acid Species	Abbreviation	RT (min)	Precursor Ion (*m*/*z*)	Product Ion (*m*/*z*)	Collision Energy (eV)	Coefficient of Determination (r^2^)
**Classical bile acids**						
Glycocholic acid	GCA	24.88	464.10	74.00	39	0.998
Taurocholic acid	TCA	25.97	514.00	124.00	51	0.997
Cholic acid	CA	25.59	407.00	343.40	33	0.997
Taurocholic acid 3-sulfate	TCA-3S	20.37	296.70	96.90	38	0.999
Cholic acid 3-sulfate	CA-3S	21.10	487.10	96.90	46	0.999
Glycochenodeoxycholic acid	GCDCA	28.87	448.30	74.00	42	0.997
Taurochenodeoxycholic acid	TCDCA	29.30	498.00	124.10	51	0.995
Chenodeoxycholic acid	CDCA	32.79	391.10	373.20	32	0.997
Glycochenodeoxycholic acid 3-sulfate	GCDCA-3S	22.90	263.80	96.90	40	0.999
Taurochenodeoxycholic acid 3-sulfate	TCDCA-3S	23.26	288.50	96.90	39	0.997
Chenodeoxycholic acid 3-sulfate	CDCA-3S	25.47	471.10	96.80	55	0.999
Glycoursodeoxycholic acid	GUDCA	29.15	448.30	73.80	42	0.994
Tauroursodeoxycholic acid	TUDCA	29.44	498.30	123.90	51	0.996
Ursodeoxycholic acid	UDCA	26.52	391.10	373.50	32	0.998
Glycoursodeoxycholic acid 3-sulfate	GUDCA-3S	18.84	263.60	96.90	40	0.999
Tauroursodeoxycholic acid 3-sulfate	TUDCA-3S	23.33	288.50	96.90	39	0.995
Ursodeoxycholic acid 3-sulfate	UDCA-3S	21.24	471.10	97.00	55	0.998
Glycodeoxycholic acid	GDCA	28.20	448.30	74.00	42	0.995
Taurodeoxycholic acid	TDCA	29.52	498.00	124.10	51	0.993
Deoxycholic acid	DCA	32.74	391.10	345.40	35	0.999
Glycodeoxycholic acid 3-sulfate	GDCA-3S	22.90	263.60	97.00	40	0.999
Taurodeoxycholic acid 3-sulfate	TDCA-3S	23.84	288.50	97.00	39	0.998
Deoxycholic acid 3-sulfate	DCA-3S	26.35	471.10	96.80	55	0.999
Glycolithocholic acid	GLCA	35.32	432.10	73.90	39	0.997
Taurolithocholic acid	TLCA	37.11	482.10	124.00	49	0.995
Lithocholic acid	LCA	43.55	375.40	375.10	10	0.999
Glycolithocholic acid 3-sulfate	GLCA-3S	25.65	255.60	97.00	40	0.997
Taurolithocholic acid 3-sulfate	TLCA-3S	27.46	280.50	96.90	37	0.998
Lithocholic acid 3-sulfate	LCA-3S	31.31	455.10	96.90	44	0.997
Glycohyocholic acid	GHCA	23.85	464.30	74.00	39	0.998
Hyocholic acid	HCA	25.08	407.20	389.10	34	0.997
**Hydroxylated bile acids**						
1β-hydroxy-glycocholic acid	GCA-1β-ol	16.35	480.20	74.00	43	0.998
1β-hydroxy-taurocholic acid	TCA-1β-ol	17.03	530.10	124.00	54	0.998
1β-hydroxy-cholic acid	CA-1β-ol	16.45	423.10	263.30	37	0.999
6α-hydroxy-glycocholic acid	GCA-6α-ol	18.90	480.20	74.00	42	0.999
6α-hydroxy-cholic acid	CA-6α-ol	19.27	423.10	313.30	37	0.999
1β-hydroxy-chenodeoxycholic acid	CDCA-1β-ol	20.07	407.20	389.30	35	0.995
**3β-hydroxy-Δ^5^ bile acids**						
3β,7α,12α-trihydroxy-5-cholenoic acid N-(carboxymethyl)amide	G-Δ^5^-3β,7α,12α-(OH)_3_	20.00	462.10	74.00	38	0.999
3β,7α,12α-trihydroxy-5-cholenoic acid N-(2-sulfoethyl) amide	T-Δ^5^-3β,7α,12α-(OH)_3_	20.80	512.10	124.00	51	0.999
3β,7α,12α-trihydroxy-5-cholenoic acid	Δ^5^-3β,7α,12α-(OH)_3_	20.72	405.20	289.30	31	0.999
3β,7α,12α-trihydroxy-5-cholenoic acid N-(2-sulfoethyl) amide 3-sulfate	T-Δ^5^-3β,7α,12α-(OH)_3_-3S	19.35	295.70	96.90	34	0.998
3β,7α,12α-trihydroxy-5-cholenoic acid 3-sulfate	Δ^5^-3β,7α,12α-(OH)_3_-3S	19.71	485.10	97.00	49	0.995
3β,7α-dihydroxy-5-cholenoic acid N-(carboxymethyl)amide	G-Δ^5^-3β,7α-(OH)_2_	23.72	446.20	74.00	38	0.998
3β,7α-dihydroxy-5-cholenoic acid N-(2-sulfoethyl) amide	T-Δ^5^-3β,7α-(OH)_2_	24.92	496.10	124.00	42	0.998
3β,7α-dihydroxy-5-cholenoic acid	Δ^5^-3β,7α-(OH)_2_	26.07	389.10	357.30	35	0.993
3β,7α-dihydroxy-5-cholenoic acid N-(carboxymethyl) amide 3-sulfate	G-Δ^5^-3β,7α-(OH)_2_-3S	21.37	262.60	97.00	24	0.999
3β,7α-dihydroxy-5-cholenoic acid N-(2-sulfoethyl) amide 3-sulfate	T-Δ^5^-3β,7α-(OH)_2_-3S	27.38	287.50	97.00	42	0.998
3β,7α-dihydroxy-5-cholenoic acid 3-sulfate	Δ^5^-3β,7α-(OH)_2_-3S	24.09	469.10	96.90	48	0.999
3β-hydroxy-5-cholenoic acid N-(carboxymethyl)amide	G-Δ^5^-3β-OH	30.14	430.40	74.00	32	0.997
3β-hydroxy-5-cholenoic acid N-(2-sulfoethyl) amide	T-Δ^5^-3β-OH	31.44	480.10	124.00	49	0.999
3β-hydroxy-5-cholenoic acid	Δ^5^-3β-OH	38.41	373.20	355.20	26	0.992
3β-hydroxy-5-cholenoic acid N-(carboxymethyl) amide 3-sulfate	G-Δ^5^-3β-OH-3S	23.47	254.90	96.90	29	0.999
3β-hydroxy-5-cholenoic acid N-(2-sulfoethyl) amide 3-sulfate	T-Δ^5^-3β-OH-3S	24.52	279.50	96.90	25	0.998
3β-hydroxy-5-cholenoic acid 3-sulfate	Δ^5^-3β-OH-3S	28.75	453.10	96.90	42	0.998
3β,12α-dihydroxy-5-cholenoic acid	Δ^5^-3β,12α-(OH)_2_	25.98	389.10	287.30	33	0.999
**3-oxo-Δ^4^ bile acids**						
7α,12α-dihydroxy-3-oxo-4-cholenoic acid N-(carboxymethyl)amide	GCA-Δ^4^-3-one	20.58	460.10	74.00	37	0.998
7α,12α-dihydroxy-3-oxo-4-cholenoic acid N-(2-sulfoethyl) amide	TCA-Δ^4^-3-one	21.45	510.10	124.00	50	0.999
7α,12α-dihydroxy-3-oxo-4-cholenoic acid	CA-Δ^4^-3-one	21.31	403.10	123.10	39	0.999
7α-hydroxy-3-oxo-4-cholenoic acid N-(carboxymethyl)amide	GCDCA-Δ^4^-3-one	25.03	444.10	74.00	35	0.999
7α-hydroxy-3-oxo-4-cholenoic acid N-(2-sulfoethyl) amide	TCDCA-Δ^4^-3-one	26.23	494.10	124.00	44	0.999
7α-hydroxy-3-oxo-4-cholenoic acid	CDCA-Δ^4^-3-one	27.60	387.10	369.30	27	0.997
12α-hydroxy-3-oxo-4,6-choladienoic acid	CA-Δ^4^,6-3-one	26.35	385.10	341.30	27	0.997
3-oxo-4,6-choladienoic acid	CDCA-Δ^4^,6-3-one	37.16	369.10	325.30	28	0.999
**Short- and long-chain bile acids**						
nor-cholic acid	nor-CA	21.60	393.20	329.30	35	0.999
3α,7α-dihydroxy-5β-cholestanoic acid	C_27_-DHCA	44.05	433.20	415.40	45	0.991
3α,7α,12α-trihydroxy-5β-cholestanoic acid	C_27_-THCA	34.92	449.20	431.30	42	0.999

*m*/*z*, mass-to-charge ratio. Isomers with identical or closely similar MRM transitions: GCDCA-3S/GDCA-3S; TCDCA-3S/TUDCA-3S/TDCA-3S; TCDCA/TUDCA/TDCA; GCDCA/GUDCA/GDCA. Their separation was not verified.

**Table 4:** Urinary bile acid profiles measured by LC–MS/MS and plasma cholesterol, cholestanol, and 7-dehydrocholesterol concentrations measured by GC–MS.

Urinary Bile Acids	Control Group	P1	P2	P3	P4	P5	P6	P7	P8	P9	P10	P11	P12
**All bile acid species**													
**Total bile acids** (µmol/mmol Cr; %)	0.59 (100.00)	32.86 (100.00)	0.72 (100.00)	0.68 (100.00)	0.99 (100.00)	1.14 (100.00)	3.28 (100.00)	9.85 (100.00)	47.87 (100.00)	113.37 (100.00)	60.99 (100.00)	30.69 (100.00)	3.53 (100.00)
**Classical bile acids** (µmol/mmol Cr; %)	0.55 (91.70)	11.68 (35.50)	0.53 (73.40)	0.34 (50.70)	0.16 (16.50)	0.74 (64.80)	1.26 (38.40)	6.78 (68.90)	46.07 (96.30)	112.67 (99.40)	59.56 (97.70)	29.30 (95.50)	3.14 (89.20)
**Hydroxylated bile acids** (µmol/mmol Cr; %)	0.03 (4.10)	0.03 (0.10)	0.01 (0.70)	0.13 (19.70)	0.17 (17.10)	—	1.56 (47.50)	0.39 (3.90)	0.16 (0.30)	0.23 (0.20)	0.59 (0.90)	0.76 (2.40)	0.01 (0.10)
**3β-hydroxy-Δ^5^ bile acids** (µmol/mmol Cr; %)	0.02 (4.70)	21.01 (63.90)	0.16 (22.10)	0.03 (4.50)	0.30 (30.20)	0.16 (13.60)	0.02 (0.70)	1.79 (18.10)	0.36 (0.70)	0.15 (0.10)	0.17 (0.30)	0.53 (1.70)	0.16 (4.50)
**3-oxo-Δ^4^ bile acids** (µmol/mmol Cr; %)	0.03 (6.90)	0.14 (0.40)	0.03 (3.80)	0.17 (25.10)	0.36 (36.20)	0.25 (21.60)	0.44 (13.40)	0.77 (7.70)	1.28 (2.70)	0.33 (0.30)	0.68 (1.10)	0.09 (0.30)	0.22 (6.20)
**Short- and long-chain bile acids** (µmol/mmol Cr; %)	0.02 (2.30)	0.01 (0.10)	—	—	—	—	—	0.15 (1.40)	—	—	—	0.01 (0.10)	0.01 (0.10)
**After omission of UDCA-assigned species**													
**Total bile acids** (µmol/mmol Cr; %)	0.08 (100.00)	32.69 (100.00)	0.28 (100.00)	0.35 (100.00)	0.97 (100.00)	0.47 (100.00)	2.14 (100.00)	5.46 (100.00)	2.89 (100.00)	32.32 (100.00)	17.30 (100.00)	8.98 (100.00)	0.77 (100.00)
**Classical bile acids** (µmol/mmol Cr; %)	0.04 (58.60)	11.50 (35.20)	0.09 (32.40)	0.02 (5.60)	0.14 (14.90)	0.07 (15.00)	0.12 (5.80)	2.38 (43.60)	1.09 (37.70)	31.61 (97.80)	15.87 (91.70)	7.59 (84.50)	0.38 (49.20)
**Hydroxylated bile acids** (µmol/mmol Cr; %)	0.03 (14.20)	0.03 (0.10)	0.01 (1.80)	0.13 (37.60)	0.17 (17.50)	—	1.56 (72.70)	0.39 (7.10)	0.16 (5.60)	0.23 (0.70)	0.58 (3.40)	0.76 (8.50)	0.01 (0.60)
**3β-hydroxy-Δ^5^ bile acids** (µmol/mmol Cr; %)	0.02 (31.80)	21.01 (64.30)	0.16 (56.20)	0.03 (8.80)	0.30 (30.80)	0.16 (32.90)	0.02 (1.10)	1.78 (32.60)	0.36 (12.50)	0.15 (0.50)	0.17 (1.00)	0.53 (5.90)	0.16 (20.80)
**3-oxo-Δ^4^ bile acids** (µmol/mmol Cr; %)	0.03 (20.50)	0.14 (0.40)	0.03 (9.60)	0.17 (48.00)	0.36 (36.90)	0.25 (52.10)	0.44 (20.40)	0.77 (14.00)	1.28 (44.10)	0.33 (1.00)	0.68 (3.90)	0.09 (1.00)	0.22 (28.50)
**Short- and long-chain bile acids** (µmol/mmol Cr; %)	0.02 (6.70)	0.01 (0.00)	—	—	—	—	—	0.14 (2.60)	—	—	—	0.01 (0.10)	0.01 (0.90)
**Cholesterol** **(mg/dL)**	210.00 ± 66.54 (*n* = 46)	211.23	272.89	255.39	161.17	171.00	*	*	255.80	159.43	*	241.35	277.07
**Cholestanol** **(µg/mL)**	2.68 ± 0.99 (*n* = 46)	5.43	1.72	7.95	4.16	3.44	*	*	2.72	3.47	*	2.77	3.96
**7-dehydrocholesterol** **(µg/mL)**	0.93 ± 0.45 (*n* = 46)	<0.30	1.84	0.63	<0.30	<0.30	*	*	0.55	0.37	*	0.49	0.67
**Diagnostic status**		HSD3B7 (A)	HSD3B7 (A)	CTX (A)	CTX (A)	CTX (A)	Unresolved	Unresolved	Biliary atresia (B)	PFIC1 (B)	Unresolved	Unresolved	Unresolved

Urinary bile acids: semi-quantitative concentrations (µmol/mmol Cr), with percentages of total bile acids in parentheses. Plasma cholesterol: mg/dL; cholestanol and 7-dehydrocholesterol: µg/mL. Patient values are individual observations. Control bile acid values are arithmetic means of included positive results; percentages were calculated individually before averaging. Control *n*/50: total 50/50, classical 50/50, hydroxylated 17/50, 3β-hydroxy-Δ^5^ 45/50, 3-oxo-Δ^4^ 19/50, and short-/long-chain 1/50. Mean percentages across these subsets need not sum to 100. Control plasma sterols are mean ± SD. 7-Dehydrocholesterol levels for P1, P4, and P5 were below the lowest GC–MS calibration standard and are reported as <0.30 µg/mL. The value of 0.30 µg/mL denotes the lowest calibration standard, not a validated lower limit of quantification. Treatment: Table 2; individual metabolites: Supplementary Table S1. Analyte-specific detection and quantification limits were not validated. The second block excludes UDCA, GUDCA, TUDCA, and their 3-sulfates; percentages use the retained total of each individual. Bold indicates bile acid group headings.* No plasma samples. A, genetically confirmed IEBAS; B, received another diagnosis; unresolved, no definitive etiological diagnosis. Cr, creatinine; *n*, included observations; P, patient; 3β-HSD, 3β-hydroxy-Δ^5^-C_27_-steroid dehydrogenase/isomerase; CTX, cerebrotendinous xanthomatosis; PFIC1, progressive familial intrahepatic cholestasis type 1; SD, standard deviation; —, no numerical group result; non-detection and below-quantification results are not distinguished.

## Data Availability

The data presented in this study are available from the corresponding author upon reasonable request.

## Supplementary Materials

The following supporting information can be downloaded at: https://www.mdpi.com/article/10.3390/metabo16100753/s1, Table S1: Individual urinary bile acid species in patients investigated for inborn errors of bile acid synthesis or unexplained cholestasis and healthy controls.

## Appendix A

**Table A1:** Bile acid species, abbreviations, and internal standards assigned to each analyte.

Abbreviation	Bile Acid Species/Chemical Name	Assigned Internal Standard
**Classical bile acids**		
GCA	Glycocholic acid	GCA-d_4_
TCA	Taurocholic acid	TCA-d_4_
CA	Cholic acid	CA-d_4_
GCDCA	Glycochenodeoxycholic acid	GCDCA-d_4_
TCDCA	Taurochenodeoxycholic acid	TCDCA-d_4_
CDCA	Chenodeoxycholic acid	CA-d_4_
GUDCA	Glycoursodeoxycholic acid	GCDCA-d_4_
TUDCA	Tauroursodeoxycholic acid	TCDCA-d_4_
UDCA	Ursodeoxycholic acid	CA-d_4_
GDCA	Glycodeoxycholic acid	GCDCA-d_4_
TDCA	Taurodeoxycholic acid	TCDCA-d_4_
DCA	Deoxycholic acid	CA-d_4_
GLCA	Glycolithocholic acid	GCDCA-d_4_
TLCA	Taurolithocholic acid	TCDCA-d_4_
LCA	Lithocholic acid	CA-d_4_
GHCA	Glycohyocholic acid	GCA-d_4_
HCA	Hyocholic acid	CA-d_4_
**Internal standards**		
CA-d_4_	Cholic acid; 2,2,4,4-d_4_	
GCA-d_4_	Glycocholic acid; 2,2,4,4-d_4_	
TCA-d_4_	Taurocholic acid sodium salt; 2,2,4,4-d_4_	
GCDCA-d_4_	Glycochenodeoxycholic acid sodium salt; 2,2,4,4-d_4_	
TCDCA-d_4_	Taurochenodeoxycholic acid sodium salt; 2,2,4,4-d_4_	
CDCA-3S-d_5_	Chenodeoxycholic acid 3-sulfate-d_5_	
GCDCA-3S-d_5_	Glycochenodeoxycholic acid 3-sulfate-d_5_	
TCDCA-3S-d_5_	Taurochenodeoxycholic acid 3-sulfate-d_5_	
**3-sulfated bile acids**		
TCA-3S	Taurocholic acid 3-sulfate	TCDCA-3S-d_5_
CA-3S	Cholic acid 3-sulfate	CDCA-3S-d_5_
GCDCA-3S	Glycochenodeoxycholic acid 3-sulfate	GCDCA-3S-d_5_
TCDCA-3S	Taurochenodeoxycholic acid 3-sulfate	TCDCA-3S-d_5_
CDCA-3S	Chenodeoxycholic acid 3-sulfate	CDCA-3S-d_5_
GUDCA-3S	Glycoursodeoxycholic acid 3-sulfate	GCDCA-3S-d_5_
TUDCA-3S	Tauroursodeoxycholic acid 3-sulfate	TCDCA-3S-d_5_
UDCA-3S	Ursodeoxycholic acid 3-sulfate	CDCA-3S-d_5_
GDCA-3S	Glycodeoxycholic acid 3-sulfate	GCDCA-3S-d_5_
TDCA-3S	Taurodeoxycholic acid 3-sulfate	TCDCA-3S-d_5_
DCA-3S	Deoxycholic acid 3-sulfate	CDCA-3S-d_5_
GLCA-3S	Glycolithocholic acid 3-sulfate	GCDCA-3S-d_5_
TLCA-3S	Taurolithocholic acid 3-sulfate	TCDCA-3S-d_5_
LCA-3S	Lithocholic acid 3-sulfate	CDCA-3S-d_5_
**Hydroxylated bile acids**		
GCA-1β-ol	1β-hydroxy-glycocholic acid	GCA-d_4_
TCA-1β-ol	1β-hydroxy-taurocholic acid	TCA-d_4_
CA-1β-ol	1β-hydroxy-cholic acid	CA-d_4_
GCA-6α-ol	6α-hydroxy-glycocholic acid	GCA-d_4_
CA-6α-ol	6α-hydroxy-cholic acid	CA-d_4_
CDCA-1β-ol	1β-hydroxy-chenodeoxycholic acid	CA-d_4_
**3β-hydroxy-Δ^5^ bile acids**		
G-Δ^5^-3β,7α,12α-(OH)_3_	3β,7α,12α-trihydroxy-5-cholenoic acid N-(carboxymethyl)amide	GCA-d_4_
T-Δ^5^-3β,7α,12α-(OH)_3_	3β,7α,12α-trihydroxy-5-cholenoic acid N-(2-sulfoethyl)amide	TCA-d_4_
Δ^5^-3β,7α,12α-(OH)_3_	3β,7α,12α-trihydroxy-5-cholenoic acid	CA-d_4_
T-Δ^5^-3β,7α,12α-(OH)_3_-3S	3β,7α,12α-trihydroxy-5-cholenoic acid N-(2-sulfoethyl)amide 3-sulfate	TCDCA-3S-d_5_
Δ^5^-3β,7α,12α-(OH)_3_-3S	3β,7α,12α-trihydroxy-5-cholenoic acid 3-sulfate	CDCA-3S-d_5_
G-Δ^5^-3β,7α-(OH)_2_	3β,7α-dihydroxy-5-cholenoic acid N-(carboxymethyl)amide	GCDCA-d_4_
T-Δ^5^-3β,7α-(OH)_2_	3β,7α-dihydroxy-5-cholenoic acid N-(2-sulfoethyl)amide	TCDCA-d_4_
Δ^5^-3β,7α-(OH)_2_	3β,7α-dihydroxy-5-cholenoic acid	CA-d_4_
G-Δ^5^-3β,7α-(OH)_2_-3S	3β,7α-dihydroxy-5-cholenoic acid N-(carboxymethyl)amide 3-sulfate	GCDCA-3S-d_5_
T-Δ^5^-3β,7α-(OH)_2_-3S	3β,7α-dihydroxy-5-cholenoic acid N-(2-sulfoethyl)amide 3-sulfate	TCDCA-3S-d_5_
Δ^5^-3β,7α-(OH)_2_-3S	3β,7α-dihydroxy-5-cholenoic acid 3-sulfate	CDCA-3S-d_5_
G-Δ^5^-3β-OH	3β-hydroxy-5-cholenoic acid N-(carboxymethyl)amide	GCDCA-d_4_
T-Δ^5^-3β-OH	3β-hydroxy-5-cholenoic acid N-(2-sulfoethyl)amide	TCDCA-d_4_
Δ^5^-3β-OH	3β-hydroxy-5-cholenoic acid	CA-d_4_
G-Δ^5^-3β-OH-3S	3β-hydroxy-5-cholenoic acid N-(carboxymethyl)amide 3-sulfate	GCDCA-3S-d_5_
T-Δ^5^-3β-OH-3S	3β-hydroxy-5-cholenoic acid N-(2-sulfoethyl)amide 3-sulfate	TCDCA-3S-d_5_
Δ^5^-3β-OH-3S	3β-hydroxy-5-cholenoic acid 3-sulfate	CDCA-3S-d_5_
Δ^5^-3β,12α-(OH)_2_	3β,12α-dihydroxy-5-cholenoic acid	CA-d_4_
**3-oxo-Δ^4^ bile acids**		
GCA-Δ^4^-3-one	7α,12α-dihydroxy-3-oxo-4-cholenoic acid N-(carboxymethyl)amide	GCA-d_4_
TCA-Δ^4^-3-one	7α,12α-dihydroxy-3-oxo-4-cholenoic acid N-(2-sulfoethyl)amide	TCA-d_4_
CA-Δ^4^-3-one	7α,12α-dihydroxy-3-oxo-4-cholenoic acid	CA-d_4_
GCDCA-Δ^4^-3-one	7α-hydroxy-3-oxo-4-cholenoic acid N-(carboxymethyl)amide	GCDCA-d_4_
TCDCA-Δ^4^-3-one	7α-hydroxy-3-oxo-4-cholenoic acid N-(2-sulfoethyl)amide	TCDCA-d_4_
CDCA-Δ^4^-3-one	7α-hydroxy-3-oxo-4-cholenoic acid	CA-d_4_
**3-oxo-Δ^4^,^6^ bile acids**		
CA-Δ^4^,^6^-3-one	12α-hydroxy-3-oxo-4,6-choladienoic acid	CA-d_4_
CDCA-Δ^4^,^6^-3-one	3-oxo-4,6-choladienoic acid	CA-d_4_
**Short- and long-chain bile acids**		
nor-CA	nor-cholic acid	CA-d_4_
C_27_-DHCA	3α,7α-dihydroxy-5β-cholestanoic acid	CA-d_4_
C_27_-THCA	3α,7α,12α-trihydroxy-5β-cholestanoic acid	CA-d_4_
