# Supplementary material for: Comprehensive Urinary Bile Acid Profiling in Patients Investigated for Inborn Errors of Bile Acid Synthesis Using LC–MS/MS
Source: Metabolites. 2026 Oct 8;16(10):753. doi: 10.3390/metabo16100753 (PMC13706915; doi:10.3390/metabo16100753)
Supplement: Supplementary file 1 [file metabolites-16-00753-s001.zip › metabolites-4573487-supplementary.pdf]

Supplementary Table S1. Individual urinary bile acid species in patients investigated for inborn errors of bile acid synthesis or unexplained cholestasis and healthy controls.

Bile acid species	Controls	Patients	P1	P2	P3	P4	P5	P6	P7	P8	P9	P10	P11	P12
**Classical bile acids**														
GCA	0.008<br>(0.001–0.046)<br>50/50 (100%)	0.058<br>(0.005–0.266)<br>8/12 (66.7%)	0.008	0.005	*	*	*	0.006	0.016	0.146	0.007	0.008	0.266	*
TCA	*<br>0/50 (0%)	0.123<br>(0.019–0.282)<br>6/12 (50%)	*	*	*	*	*	0.019	0.034	0.023	0.282	0.227	0.155	*
CA	0.002<br>(0.001–0.004)<br>5/50 (10%)	0.008<br>(0.007–0.009)<br>2/12 (16.7%)	0.009	*	*	*	*	*	0.007	*	*	*	*	*
TCA-3S	0.010<br>1/50 (2%)	0.075<br>(0.069–0.083)<br>3/12 (25%)	0.069	*	*	*	*	*	0.083	*	0.074	*	*	*
CA-3S	0.004<br>(0.001–0.016)<br>12/50 (24%)	0.013<br>(0.001–0.033)<br>8/12 (66.7%)	0.033	0.001[†]	0.003	0.02	0.019	*	0.014	*	*	0.01	0.004	*
GCDCA	0.001<br>(0.001–0.005)<br>50/50 (100%)	2.813<br>(0.001–21.484)<br>10/12 (83.3%)	0.001[†]	0.001[†]	*	*	0.007	0.002	0.014	0.036	21.484	6.561	0.023	0.002
TCDCA	*<br>0/50 (0%)	0.582<br>(0.005–0.999)<br>3/12 (25%)	*	*	*	*	*	*	*	*	0.743	0.999	0.005	*
CDCA	*<br>0/50 (0%)	*<br>0/12 (0%)	*	*	*	*	*	*	*	*	*	*	*	*
GCDCA-3S	*<br>0/50 (0%)	0.271<br>(0.045–0.819)<br>4/12 (33.3%)	0.819	*	*	*	*	*	0.045	0.061	*	0.157	*	*
TCDCA-3S	0.013<br>(0.005–0.022)<br>6/50 (12%)	0.393<br>(0.004–2.073)<br>6/12 (50%)	2.073	0.004	*	*	*	*	0.072	0.099	*	0.059	*	0.05
CDCA-3S	0.006<br>(0.001–0.019)<br>16/50 (32%)	0.008<br>(0.001–0.025)<br>10/12 (83.3%)	0.003	0.001	0.003	0.025	0.005	0.008	0.02	0.004	*	*	0.009	0.002
GUDCA	0.515<br>(0.050–2.011)<br>50/50 (100%)	18.247<br>(0.165–80.857)<br>11/12 (91.7%)	0.165	**0.433**	0.321	*	0.662	1.13	4.38	**44.975**	**80.857**[‡]	**43.342**	**21.698**	**2.757**
TUDCA	*<br>0/50 (0%)	0.181<br>(0.005–0.348)<br>3/12 (25%)	*	*	*	*	*	*	*	*	0.191	0.348	0.005	*
UDCA	*<br>0/50 (0%)	*<br>0/12 (0%)	*	*	*	*	*	*	*	*	*	*	*	*

Bile acid species	Controls	Patients	P1	P2	P3	P4	P5	P6	P7	P8	P9	P10	P11	P12
GUDCA-3S	*<br>0/50 (0%)	0.003<br>1/12 (8.3%)	0.003	*	*	*	*	*	*	*	*	*	*	*
TUDCA-3S	*<br>0/50 (0%)	*<br>0/12 (0%)	*	*	*	*	*	*	*	*	*	*	*	*
UDCA-3S	0.002<br>(0.001–0.007)<br>14/50 (28%)	0.007<br>(0.001–0.018)<br>8/12 (66.7%)	0.008	0.001[†]	0.002	0.018	0.006	0.005	0.015	*	*	*	0.004	*
GDCA	0.001<br>(0.001–0.002)<br>25/50 (50%)	2.059<br>(0.001–8.132)<br>7/12 (58.3%)	0.001[†]	0.001[†]	*	*	*	*	0.002	0.038	8.132	6.219	0.019	*
TDCA	0.001<br>1/50 (2%)	0.326<br>(0.002–0.536)<br>3/12 (25%)	*	*	*	*	*	*	*	*	0.44	0.536	0.002	*
DCA	*<br>0/50 (0%)	*<br>0/12 (0%)	*	*	*	*	*	*	*	*	*	*	*	*
GDCA-3S	0.008<br>1/50 (2%)	1.240<br>(0.009–3.624)<br>3/12 (25%)	3.624	*	*	*	*	*	*	*	*	0.086	0.009	*
TDCA-3S	0.021<br>(0.011–0.033)<br>7/50 (14%)	0.822<br>(0.020–4.840)<br>7/12 (58.3%)	4.840	0.02	*	0.077	*	*	0.186	0.252	*	0.251	*	0.13
DCA-3S	0.005<br>(0.001–0.018)<br>17/50 (34%)	0.006<br>(0.002–0.018)<br>11/12 (91.7%)	0.002	*	0.002[†]	0.007	0.004	0.006	0.018	0.003	0.004	0.006	0.008	0.003
GLCA	*<br>0/50 (0%)	*<br>0/12 (0%)	*	*	*	*	*	*	*	*	*	*	*	*
TLCA	*<br>0/50 (0%)	0.008<br>1/12 (8.3%)	*	*	*	*	*	*	*	*	0.008	*	*	*
LCA	0.002<br>(0.001–0.012)<br>50/50 (100%)	0.033<br>(0.002–0.174)<br>12/12 (100%)	0.002	0.004	0.007	0.013	0.022	0.06	0.174	0.026	0.044	0.02	0.005	0.014
GLCA-3S	0.003<br>1/50 (2%)	0.215<br>(0.003–0.558)<br>3/12 (25%)	0.003	*	*	*	*	*	0.558	*	*	*	*	0.085
TLCA-3S	0.005<br>1/50 (2%)	0.272<br>(0.005–1.094)<br>6/12 (50%)	0.005	0.009	*	*	*	*	1.094	0.33	*	0.11	*	0.086
LCA-3S	0.001<br>(0.001–0.004)<br>24/50 (48%)	0.003<br>(0.001–0.007)<br>11/12 (91.7%)	0.001	0.001[†]	*	0.002	0.003	0.005	0.007	0.005	0.002	0.005	0.004	0.003
GHCA	0.017<br>(0.001–0.105)<br>50/50 (100%)	0.126<br>(0.005–0.612)<br>11/12 (91.7%)	0.009	0.044	0.005	*	0.011	0.018	0.038	0.068	0.394	0.612	0.186	0.005

Bile acid species	Controls	Patients	P1	P2	P3	P4	P5	P6	P7	P8	P9	P10	P11	P12
HCA	*<br>0/50 (0%)	6.899<br>1/12 (8.3%)	*	*	*	*	*	*	*	*	*	*	6.899	*
**TOTAL**			**11.678**	**0.525**	**0.343**	**0.162**	**0.739**	**1.259**	**6.777**	**46.066**	**112.66**	**59.556**	**29.301**	**3.137**
**Hydroxylated bile acids**														
GCA-1β-ol	*<br>0/50 (0%)	0.007<br>(0.005–0.009)<br>2/12 (16.7%)	*	*	*	*	*	*	*	*	0.005	*	0.009	*
TCA-1β-ol	*<br>0/50 (0%)	0.340<br>(0.007–1.450)<br>7/12 (58.3%)	0.007	*	*	**0.149**	*	**1.45**	0.253	*	0.160	0.099	0.264	*
CA-1β-ol	*<br>0/50 (0%)	*<br>0/12 (0%)	*	*	*	*	*	*	*	*	*	*	*	*
GCA-6α-ol	0.017<br>(0.001–0.115)<br>17/50 (34%)	0.093<br>(0.005–0.491)<br>11/12 (91.7%)	0.012	0.005	0.005	0.02	*	0.107	0.133	**0.163**	0.064	0.017	**0.491**	0.005
CA-6α-ol	0.027<br>(0.014–0.044)<br>4/50 (8%)	0.013<br>1/12 (8.3%)	0.013	*	*	*	*	*	*	*	*	*	*	*
CDCA-1β-ol	0.040<br>1/50 (2%)	0.298<br>(0.128–0.467)<br>2/12 (16.7%)	*	*	**0.128**	*	*	*	*	*	*	0.467	*	*
**TOTAL**			**0.032**	**0.005**	**0.133**	**0.169**	*	**1.557**	**0.386**	**0.163**	**0.229**	**0.583**	**0.764**	**0.005**
**3β-hydroxy-Δ^5 bile acids**														
G-Δ^5-3β,7α,12α-$(OH)_3$	0.003<br>(0.001–0.008)<br>9/50 (18%)	0.025<br>(0.002–0.090)<br>5/12 (41.7%)	0.09	0.003	*	0.022	*	*	*	*	*	*	0.002	0.01
T-Δ^5-3β,7α,12α-$(OH)_3$	0.005<br>(0.004–0.005)<br>2/50 (4%)	0.010<br>(0.005–0.014)<br>2/12 (16.7%)	*	*	*	*	0.014	*	*	*	*	*	0.005	*
Δ^5-3β,7α,12α-$(OH)_3$	0.010<br>1/50 (2%)	0.951<br>(0.085–1.816)<br>2/12 (16.7%)	**1.816**	*	*	*	*	*	0.085	*	*	*	*	*
T-Δ^5-3β,7α,12α-$(OH)_3$-3S	*<br>0/50 (0%)	0.063<br>(0.051–0.075)<br>2/12 (16.7%)	0.051	*	*	*	*	*	*	0.075	*	*	*	*
Δ^5-3β,7α,12α-$(OH)_3$-3S	0.005<br>(0.001–0.018)<br>16/50 (32%)	0.045<br>(0.012–0.130)<br>8/12 (66.7%)	0.026	*	*	0.13	0.045	*	0.02	0.076	0.033	*	0.012	0.014
G-Δ^5-3β,7α-$(OH)_2$	0.006<br>(0.001–0.026)<br>6/50 (12%)	1.103<br>(0.003–4.394)<br>4/12 (33.3%)	**4.394**	*	*	0.005	0.008	*	*	*	*	*	0.003	*

**Bile acid species**	**Controls**	**Patients**	**P1**	**P2**	**P3**	**P4**	**P5**	**P6**	**P7**	**P8**	**P9**	**P10**	**P11**	**P12**
T-Δ^5-3β,7α-$(OH)_2$	0.003<br>(0.001–0.008)<br>5/50 (10%)	0.015<br>(0.002–0.024)<br>4/12 (33.3%)	0.011	*	*	0.024	0.023	*	*	*	*	*	0.002	*
Δ^5-3β,7α-$(OH)_2$	*<br>0/50 (0%)	0.057<br>1/12 (8.3%)	0.057	*	*	*	*	*	*	*	*	*	*	*
G-Δ^5-3β,7α-$(OH)_2$-3S	*<br>0/50 (0%)	0.019<br>(0.005–0.032)<br>2/12 (16.7%)	*	*	*	*	*	*	0.032	*	*	*	0.005	*
T-Δ^5-3β,7α-$(OH)_2$-3S	0.002<br>1/50 (2%)	*<br>0/12 (0%)	*	*	*	*	*	*	*	*	*	*	*	*
Δ^5-3β,7α-$(OH)_2$-3S	0.002<br>(0.001–0.009)<br>20/50 (40%)	1.329<br>(0.001–14.549)<br>11/12 (91.7%)	**14.549**	$0.001^{\dagger}$	0.002	0.031	$0.002^{\dagger}$	0.004	0.007	0.01	0.005	0.004	0.002	*
G-Δ^5-3β-OH	0.001<br>(0.001–0.003)<br>19/50 (38%)	0.003<br>(0.001–0.006)<br>9/12 (75%)	*	$0.001^{\dagger}$	0.002	0.003	0.003	0.005	0.004	0.005	0.006	*	0.001	*
T-Δ^5-3β-OH	*<br>0/50 (0%)	0.045<br>(0.011–0.078)<br>2/12 (16.7%)	*	*	*	*	*	*	0.078	*	*	*	0.011	*
Δ^5-3β-OH	*<br>0/50 (0%)	0.481<br>(0.001–1.346)<br>4/12 (33.3%)	$0.001^{\dagger}$	**0.141**	*	*	*	*	**1.346**	*	*	*	0.434	*
G-Δ^5-3β-OH-3S	0.018<br>(0.002–0.122)<br>38/50 (76%)	0.047<br>(0.007–0.098)<br>7/12 (58.3%)	0.007	*	0.011	**0.069**	**0.045**	*	0.057	*	0.098	*	0.042	*
T-Δ^5-3β-OH-3S	0.002<br>(0.001–0.005)<br>7/50 (14%)	0.057<br>(0.005–0.193)<br>12/12 (100%)	0.005	0.010	0.007	0.007	0.013	0.006	0.141	0.193	0.011	0.161	0.006	**0.127**
Δ^5-3β-OH-3S	0.003<br>(0.001–0.010)<br>19/50 (38%)	0.006<br>(0.002–0.011)<br>11/12 (91.7%)	0.004	0.002	0.009	0.007	0.003	0.008	0.011	0.004	*	0.008	0.004	0.01
Δ^5-3β,12α-$(OH)_2$	*<br>0/50 (0%)	*<br>0/12 (0%)	*	*	*	*	*	*	*	*	*	*	*	*
**TOTAL**			**21.011**	**0.158**	**0.031**	**0.298**	**0.156**	**0.023**	**1.781**	**0.363**	**0.153**	**0.173**	**0.529**	**0.161**
**3-oxo-Δ^4 bile acids**														
GCA-Δ^4-3-one	0.003<br>1/50 (2%)	0.009<br>(0.001–0.034)<br>6/12 (50%)	0.002	*	0.003	*	0.005	*	*	*	0.006	0.034	0.001	*
TCA-Δ^4-3-one	0.010<br>(0.001–0.040)<br>6/50 (12%)	0.098<br>(0.005–0.240)<br>3/12 (25%)	*	*	*	*	*	*	0.048	0.240	*	*	0.005	*

Bile acid species	Controls	Patients	P1	P2	P3	P4	P5	P6	P7	P8	P9	P10	P11	P12
CA-Δ^4-3-one	0.004<br>(0.002–0.007)<br>4/50 (8%)	0.013<br>(0.001–0.025)<br>6/12 (50%)	0.001†	*	0.004	*	0.015	*	*	0.025	*	0.025	0.007	*
GCDCA-Δ^4-3-one	0.002<br>(0.001–0.003)<br>2/50 (4%)	0.036<br>(0.002–0.107)<br>4/12 (33.3%)	0.006	*	*	*	*	*	*	0.107	*	0.028	0.002	*
TCDCA-Δ^4-3-one	0.003<br>(0.002–0.004)<br>2/50 (4%)	0.055<br>(0.005–0.151)<br>4/12 (33.3%)	*	*	*	0.05	0.013	*	0.151	*	*	*	0.005	*
CDCA-Δ^4-3-one	0.047<br>(0.003–0.136)<br>9/50 (18%)	0.237<br>1/12 (8.3%)	*	*	*	*	*	*	0.237	*	*	*	*	*
CA-Δ^4,6-3-one	0.002<br>1/50 (2%)	0.298<br>(0.027–0.904)<br>12/12 (100%)	0.124	0.027	**0.163**	**0.307**	**0.214**	**0.437**	0.223	**0.904**	**0.322**	**0.589**	0.045	**0.220**
CDCA-Δ^4,6-3-one	0.004<br>(0.003–0.004)<br>4/50 (8%)	0.043<br>(0.002–0.106)<br>3/12 (25%)	0.002†	*	*	*	*	*	0.106	*	*	*	0.022	*
**TOTAL**			**0.135**	**0.027**	**0.17**	**0.357**	**0.247**	**0.437**	**0.765**	**1.276**	**0.328**	**0.676**	**0.087**	**0.22**
**Short- and long-chain bile acids**														
nor-CA	0.006<br>1/50 (2%)	0.007<br>(0.002–0.012)<br>3/12 (25%)	*	*	*	*	*	*	0.012	*	*	*	0.002†	0.007
C_{27}-DHCA	0.009<br>1/50 (2%)	0.033<br>(0.006–0.059)<br>2/12 (16.7%)	*	*	*	*	*	*	0.059	*	*	*	0.006	*
C_{27}-THCA	*<br>0/50 (0%)	0.040<br>(0.006–0.073)<br>2/12 (16.7%)	0.006	*	*	*	*	*	0.073	*	*	*	*	*

Concentrations are expressed as µmol/mmol creatinine and reported as semiquantitative estimates. The Controls and Patients columns present the arithmetic mean, minimum–maximum range, and frequency of included positive numerical results, n/N (%). Here, n is the number of participants contributing a positive numerical result, and N is the total number of controls (50) or patients (12). P1–P12 show individual patient concentrations. When n = 1, the single value is presented without a range; when n = 0, * and 0/N (0%) are shown. Entries without an included positive numerical result were excluded from summary calculations without imputation. The reported ranges describe the included observations. Bold values highlight selected species discussed in the patient profiles.

* indicates that no positive numerical result was included; this notation does not distinguish non-detection from a result below the quantification limit. Cr, creatinine; P, patient. Bile acid abbreviations and analyte-specific internal standards are listed in Appendix Table A.1. Analytical conditions and isomer-selectivity considerations are described in the main manuscript.

† Individual patient result below the lowest LC–MS/MS calibrator (<50 pmol/mL); ‡ individual patient result above the highest calibrator (>1,000 pmol/mL), based on concentrations before creatinine normalization. Chromatographic separation of GUDCA, GCDCA, and GDCA was not verified; consequently, contributions from incompletely resolved isomers to the reported GUDCA values cannot be excluded. This uncertainty affects interpretation of the GUDCA-assigned concentrations and the totals and percentages recalculated after their omission.
